# The KH domain facilitates the substrate specificity and unwinding processivity of DDX43 helicase

**DOI:** 10.1074/jbc.RA120.015824

**Published:** 2020-11-23

**Authors:** Manisha Yadav, Ravi Shankar Singh, Daniel Hogan, Venkatasubramanian Vidhyasagar, Shizhuo Yang, Ivy Yeuk Wah Chung, Anthony Kusalik, Oleg Y. Dmitriev, Miroslaw Cygler, Yuliang Wu

**Affiliations:** 1 Department of Biochemistry, Microbiology and Immunology, University of Saskatchewan, Saskatoon, Saskatchewan, Canada; 2 Department of Computer Science, University of Saskatchewan, Saskatoon, Saskatchewan, Canada

**Keywords:** KH domain, DDX43, substrate specificity, helicase processivity, SELEX, ChIP-seq, CLIP-seq, NMR, BSA, bovine serum albumin, ChIP, chromatin immunoprecipitation, CLIP, crosslinking immunoprecipitation, CML, chronic myeloid leukemia, EMSA, electrophoretic mobility shift assays, GO, gene ontology, HAGE, helicase antigen gene, hnRNP K, heterogeneous nuclear ribonucleoprotein K, HSQC, heteronuclear single quantum coherence, KH, K-homology, MEME, Multiple Expectation maximization for Motif Elicitation, Ni-NTA, nickel-nitrilotriacetic acid, PCBP1, poly(C) binding protein 1, SELEX, systematic evolution of ligands by exponential enrichment

## Abstract

The K-homology (KH) domain is a nucleic acid–binding domain present in many proteins. Recently, we found that the DEAD-box helicase DDX43 contains a KH domain in its N-terminus; however, its function remains unknown. Here, we purified recombinant DDX43 KH domain protein and found that it prefers binding ssDNA and ssRNA. Electrophoretic mobility shift assay and NMR revealed that the KH domain favors pyrimidines over purines. Mutational analysis showed that the GXXG loop in the KH domain is involved in pyrimidine binding. Moreover, we found that an alanine residue adjacent to the GXXG loop is critical for binding. Systematic evolution of ligands by exponential enrichment, chromatin immunoprecipitation–seq, and cross-linking immunoprecipitation–seq showed that the KH domain binds C-/T-rich DNA and U-rich RNA. Bioinformatics analysis suggested that the KH domain prefers to bind promoters. Using ^15^N-heteronuclear single quantum coherence NMR, the optimal binding sequence was identified as TTGT. Finally, we found that the full-length DDX43 helicase prefers DNA or RNA substrates with TTGT or UUGU single-stranded tails and that the KH domain is critically important for sequence specificity and unwinding processivity. Collectively, our results demonstrated that the KH domain facilitates the substrate specificity and processivity of the DDX43 helicase.

Helicases are molecular motors that transduce the chemical energy generated by ATP hydrolysis into oligonucleotide strand separation and protein displacement. They are involved in virtually all aspects of nucleic acid metabolism, including replication, repair, recombination, transcription, chromosome segregation, and telomere maintenance (1, 2, 3). Based on the substrate, helicases can be classified as DNA or RNA helicases, although some can function on both DNA and RNA molecules (4). According to their conserved motifs, helicases are also classified into six superfamilies, among which SF2 is the largest superfamily that includes DEAD-box helicases (5). The DEAD-box helicases usually contain 12 sequence motifs (Q, I, Ia, Ib, Ic, II, III, IV, IVa, V, Va, and VI) that are clustered in a region of 200 to 700 amino acids called the helicase core domain, which is formed by two RecA-like domains (2). Some helicases also contain accessory domain(s) at their N- or C-terminus, such as the nuclease domain, protein–protein interaction domain, and nucleic acid–binding domain.

The K-homology (KH) domain was first identified in the human heterogeneous nuclear ribonucleoprotein K (hnRNP K) 2 decades ago (6). Since then, the KH domain has been identified as a nucleic acid recognition motif in proteins in archaea, bacteria, and eukaryotes (7, 8). There are two types of KH-domains: the type I KH fold present in eukaryotes, often in multiple copies, and the type II KH fold present in prokaryotes, often in a single copy. The KH domain is approximately 70 amino acids long, with the most conserved consensus sequence VIGXXGXXI mapping to the middle of the domain. The two types of KH domains share similar secondary structure elements but have different connectivity. The KH domain is composed of three α-helices packed onto the surface of a central antiparallel β-sheet (9). Both type I and type II KH domains share a minimal βααβ core, with two additional α and β secondary structure elements positioned at either the C-terminus (type I) or the N-terminus (type II) of this core motif. The highly conserved GXXG motif forms a loop that contacts the nucleic acid and is essential for the biochemical function of the protein (8).

The typical function of KH domains is recognition of an ssRNA or ssDNA (Table S1). For example, NuSA KH1 and KH2 and Nova KH3 bind ssRNA in adenine-rich regions (10). The hnRNP K and poly-(C)–binding proteins (PCBPs) bind single-stranded cytosine-rich RNA or DNA through their KH domains (11, 12). The two KH domains of KH-type splicing regulatory protein (KSRP) bind to AU-rich sequences (13). A single KH domain can bind nucleic acids, whereas multiple KH domains function cooperatively. KH domain(s) can also function together with other domains present in the same protein. For example, the single KH domain in ankyrin repeat and KH domain-containing 1 (ANKHD1) binds to miRNAs (*i.e.*, miR-29a, miR-205, and miR-196a) (14). Multiple KH domains within a protein often lead to high affinity and specificity toward RNA targets, for instance, the two KH domains (KH1-2) of NusA (10) and the four KH domains (KH2-5) in GLD-3 (15). The KH domain can cooperate with other domain(s). For example, the KH domain and the QUA2 domain synergistically interact with RNA in QKI (Quaking homolog, KH domain containing RNA binding) (16). On the other hand, different KH domains in the same protein may have different sequence preferences and affinity, such as the four KH domains in KSRP (17) and the 14 domains in vigilin (18). In addition to nucleic acid binding, the KH domain in PINA mediates the interactions of PINA with Hjm and Hjc and regulates the hexameric assembly of PINA (19).

Humans have 40 KH domain–containing proteins (20) (Fig. S1), *Arabidopsis* 26 (21), and *Drosophila* 27 (22), and these proteins are involved in various cellular processes (7, 8) including transcription (23), post-transcriptional gene regulation (24), translation (25), and cell signaling (26). More importantly, many KH domain–containing proteins perform multiple functions (7, 8). Although KH domains have been found in numerous proteins, only two human helicases contain a KH domain, and the function of the KH domain in these two helicases is not known.

One such KH domain–containing helicase is DDX43, also known as *HAGE* (*h*elicase *a*ntigen *ge*ne), which was first identified as a cancer/testis antigen gene in a human sarcoma cell line (27). The gene is located on chromosome 6 (6q12-q13) and encodes a 73-kDa protein that belongs to the DEAD-box family of ATP-dependent RNA helicases. *DDX43* mRNA is highly enriched in a wide range of tumors, with at least 100-fold higher than in the corresponding normal tissues (27). At the protein level, DDX43 has also been detected at different levels in a variety of tumor tissues including the bladder, brain, breast, colon, esophagus, kidney, liver, lung, stomach, and small intestine, whereas none or very little protein was found in the normal tissues (28). DDX43 is also overexpressed in more than 50% of chronic myeloid leukemia (CML) cases, 20% of acute myeloid leukemia (29) cases, and more than 40% of multiple myeloma cases. High levels of DDX43 overexpression in various tumors suggest it is a potential target molecule for cancer therapy.

Belonging to the DEAD-box helicases, DDX43 possesses nucleic acid unwinding activity as reported by our group (30) and others (31, 32). DDX43 consists of a helicase core domain located at its C-terminus and a KH domain at its N-terminus (8). We have previously reported that the KH domain crucially contributes to DDX43 binding to the nucleic acids. The full-length DDX43 protein, with a single amino acid mutation in the KH domain (G84D), displayed reduced unwinding and binding activities on RNA and DNA substrates, suggesting that the KH domain is required for the full unwinding activity of DDX43 (30). Recently, another group also reported that all domains present in DDX43, including the KH domain, the helicase domain, and the connecting region between the KH and helicase domains, are required for DDX43’s helicase activity (32). However, the exact role of the KH domain in DDX43 remains unknown; therefore, we set out to characterize the properties of the KH domain of DDX43.

## Results

### DDX43 KH domain binds ssDNA and ssRNA but not blunt-end dsDNA or dsRNA

**Figure 1:**
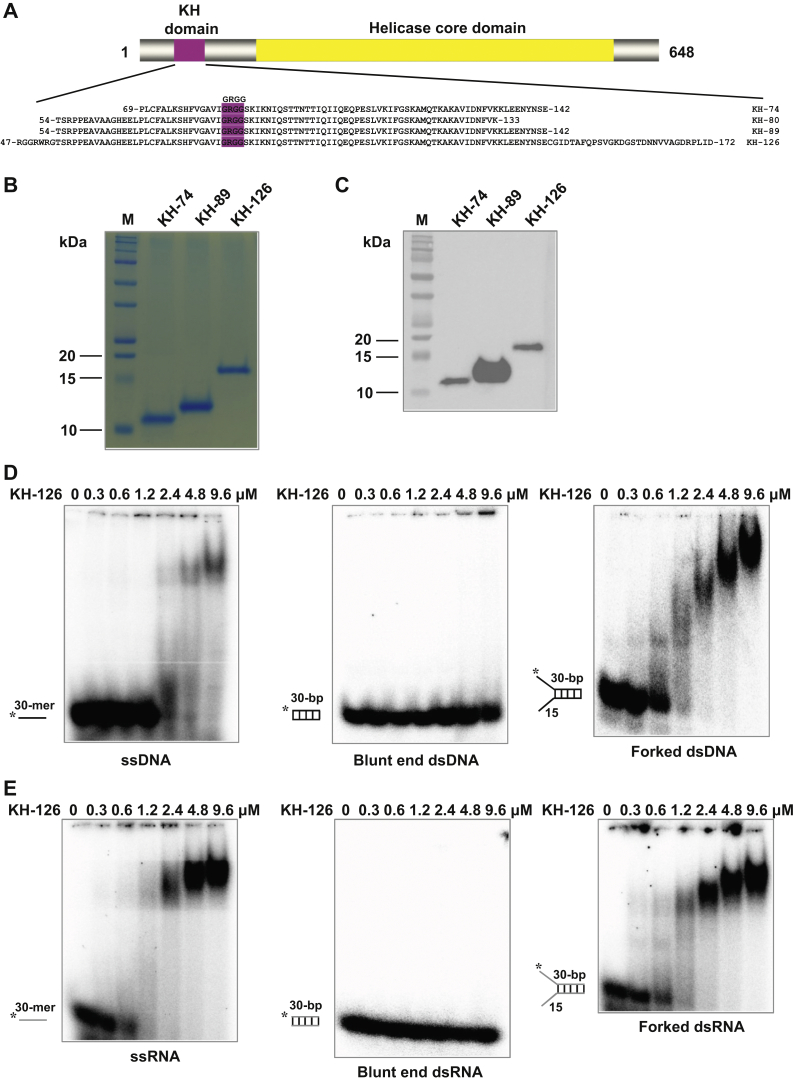
**Purification and characterization of DDX43 KH-domain proteins.***A*, the schematic representation of four DDX43 KH-domain fragments. The KH domain and GRGG loop are indicated in *red* and helicase core domain in *yellow*. *B*, the SDS-PAGE analysis of recombinant DDX43 KH-domain proteins eluting from a Sephacryl S-100 HR column. *C*, the Western blot analysis for proteins shown in panel *B* with an anti-His antibody (SC-8036, Santa Cruz). *D*–*E*, the representative EMSA images of increasing DDX43 KH-domain protein (126 aa, 0–9.6 μM) binding with 0.5 nM of indicated DNA (*D*) and RNA (*E*) substrates. See Table S2 for substrate sequence. DNA is in *black* and RNA in *gray*. EMSA, electrophoretic mobility shift assay; HR, high-resolution.

Based on the sequence homology of the DDX43 KH domain to the KH domains with known three-dimensional structures (8), we designed four constructs of DDX43 KH domain with different lengths, each containing the predicted core KH domain and named KH-74, KH-80, KH-89, and KH-126 (Fig. 1*A*). Each fragment was cloned into a pET28a vector, overexpressed in *Escherichia coli* Rosetta pLys strain and purified using nickel-nitrilotriacetic acid (Ni-NTA) affinity and Sephacryl S-100 size-exclusion chromatography. We successfully purified the KH-74, KH-89, and KH-126 proteins (Fig. 1*B*; Fig. S2, *A*–*C*), but the KH-80 protein was insoluble as revealed by the SDS-PAGE and Western blot (Fig. S2*D*). The identity of the KH domain proteins was confirmed by the Western blot analysis using an anti-His antibody (Fig. 1*C*). According to the molecular mass standards used to calibrate the size-exclusion column, the mass of KH-74, KH-89, and KH-126 proteins was 9.6 kDa (predicted from amino acid sequence as 10.4 kDa), 16.9 kDa (predicted 12.0 kDa), and 30.1 kDa (predicted 15.8 kDa), respectively, indicating that the KH-74 and KH-89 proteins exist as monomers in the solution, whereas the KH-126 forms dimers.

Because the KH domain is well known to bind ssDNA or ssRNA (8), we performed the electrophoretic mobility shift assay (EMSA) and found that the DDX43 KH-126 protein bound ssDNA and ssRNA but not blunt-end dsDNA or dsRNA (Fig. 1, *D*–*E*). Similar results were obtained for the KH-74 and KH-89 proteins (Fig. S3). In addition, we performed filter-binding assays for the DDX43 KH-126 protein (Fig. S4) and determined that its dissociation constant (*Kd*) is 1.41 ± 0.53 and 1.29 ± 0.40 μM for ssDNA and forked dsDNA and 3.01 ± 0.77 and 2.50 ± 0.69 μM for ssRNA and forked dsRNA, respectively, whereas the *Kd* for blunt-end DNA or RNA could not be determined because of their weak binding. The KH domain can bind forked duplex DNA or RNA, which might be due to the single-stranded tail present in these substrates.

### The DDX43 KH domain prefers to bind pyrimidines over purines

**Figure 2:**
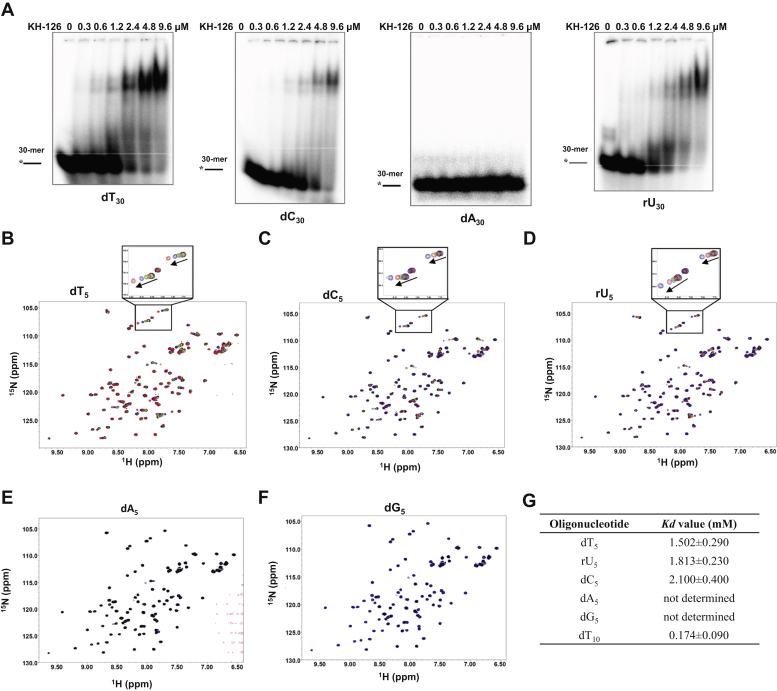
**DDX43 KH domain prefers to bind pyrimidines.***A*, the representative images of electrophoretic mobility shift assays (EMSA) performed by incubating 0.5 nM of indicated oligonucleotide with increasing DDX43 KH-domain proteins (126 aa, 0–9.6 μM) at RT for 30 min. DNA is in *black* and RNA in *gray*. *B*–*F*, ^1^H-^15^N HSQC spectrum of DDX43 KH-domain protein (89 aa, 200 μM) with indicated oligonucleotide (ranged 40–1000 μM). Chemical shifts are indicated by *arrows* in the enlarged image in panels *B*–*D*. *G*, affinities of DDX43-KH domain (89 aa) with indicated oligo determined by NMR spectroscopy. HSQC, heteronuclear single quantum coherence.

The typical function of KH domains is to bind ssRNA or ssDNA in a sequence-specific manner (8). To determine whether the DDX43 KH domain has a preference for adenine, cytosine, thymine, or uracil, we performed the EMSA with labeled dT_30_, dC_30,_ dA_30_, and rU_30_ (dG_30_ could not be synthesized), and found that the KH-126 protein has binding affinity in the order rU_30_ > dT_30_ > dC_30_ > dA_30_ (Fig. 2*A*). In addition, we performed filter-binding assays (Fig. S4) and determined that the *Kd* values for rU_30_, dT_30_, and dC_30_ are 1.48 ± 0.32, 2.45 ± 0.41, and 2.61 ± 0.56 μM, respectively, whereas the *Kd* for dA_30_ was not determined because of its weak binding. This is consistent with our previous findings using the 74 amino acid residues version of the KH domain (30). Similar results were obtained with the KH-89 protein.

To quantify the KH domain binding affinity for oligonucleotides, we used ^1^H-^15^N heteronuclear single quantum coherence (HSQC) NMR spectroscopy. Purified KH-74 protein aggregated at concentrations above 4 mg/ml. The spectra of KH-126 protein revealed partial aggregation and misfolding (Fig. S5*A*). By comparison, KH-89 protein was well folded and sufficiently stable at higher concentrations to conduct oligonucleotide binding studies by NMR (Fig. S5, *B*–*C*). Spectral quality of the KH-89 protein improved further on removal of the hexa-histidine tag (Fig. 2, *B*–*F*; Fig. S6).

We next performed titration studies with a constant concentration of KH-89 protein and increasing oligonucleotide. With successive additions of 5-mer oligonucleotides, many residues showed prominent chemical shift changes for dT_5_ (Fig. 2*B*), dC_5_ (Fig. 2*C*), and rU_5_ (Fig. 2*D*), but negligible changes for dA_5_ (Fig. 2*E*) or dG_5_ (Fig. 2*F*), indicating that the DDX43 KH domain favors binding to pyrimidines rather than purines. We then calculated the *Kd* values for dT_5_, dC_5_, and rU_5_ as 1.5 ± 0.3, 2.1 ± 0.4, and 1.8 ± 0.2 mM, respectively (Fig. 2*G*). These results suggest that pyrimidines are preferred by the KH domain of DDX43. However, no significant preference was observed within the pyrimidines.

### The GRGG loop in the KH domain is involved in nucleotide binding

**Figure 3:**
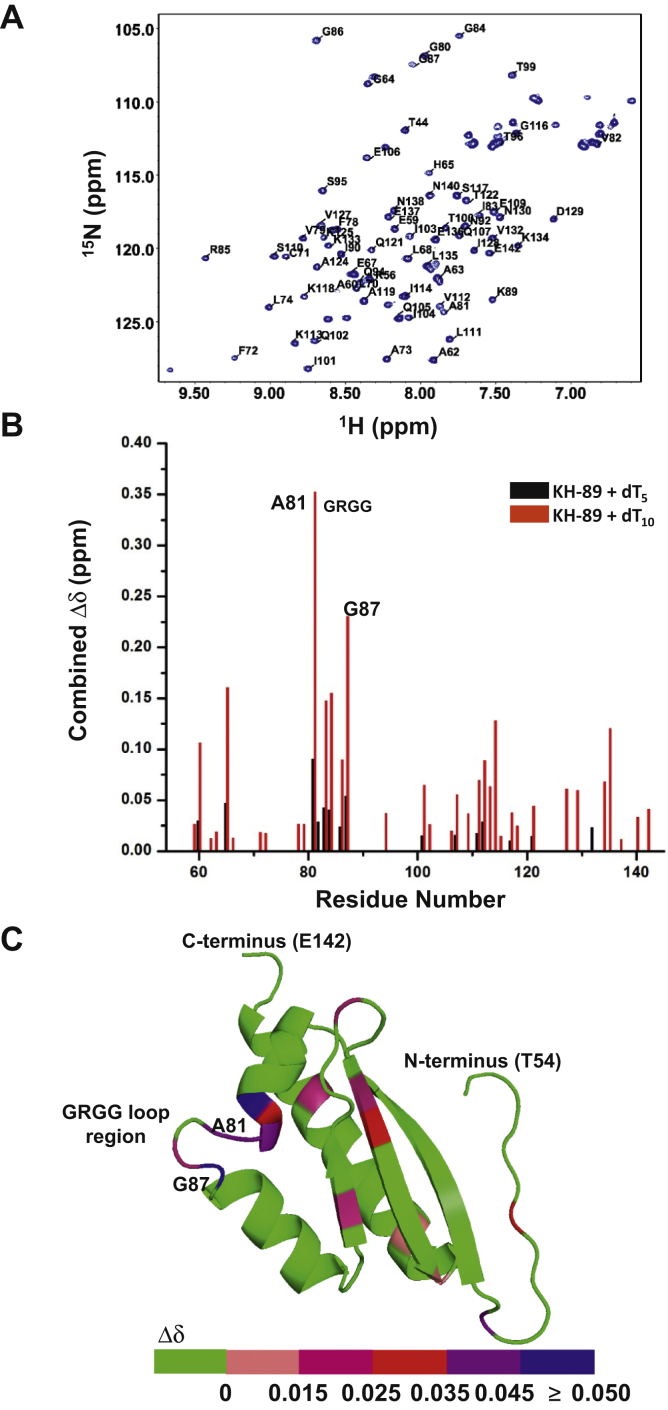
**The conserved GRGG loop region is involved in nucleotide binding.***A*, a 600-MHz ^1^H-^15^N HSQC spectrum of DDX43 KH-89 protein with some of the peak assignments. *B*, combined chemical shift changes caused by dT_5_ and dT_10_ binding to DDX43-KH domain (89 aa) as a function of amino acid. *C*, a model structure of DDX43 KH domain (89 aa) generated by Phyre 2. Potential amino acids involved in dT_5_ binding are indicated by colors according to their combined Δδ. HSQC, heteronuclear single quantum coherence.

To identify amino acids involved in nucleotide binding, a protein structure model for the DDX43 KH domain (89aa) was generated by the Phyre 2 server (33), with 98% of residues modeled at >90% confidence based on the top-ranked KH-domain template (Protein Data Bank ID 1WE8). This protein model was used for sequential amino acid peak assignment using SPARTA+ (34) (Fig. 3*A*). Eighty of 89 amino acid residues were uniquely assigned. Amino acid residues involved in nucleotide binding were identified by plotting the combined chemical shift (Δδ) as a function of the residue number (Fig. 3*B*). The pattern of chemical shift changes indicated that the GRGG loop (84–87 aa) and the adjacent amino acid residues contact the nucleic acid (Fig. 3*C*), and all the oligonucleotides bind to the same binding sites within the KH domain (Fig. S7, *A*–*D*). Among the affected residues, Ala81 and Gly87 exhibited the highest combined chemical shift change. As expected, Gly87 located in the GRGG loop is conserved in all the KH domains. However, Ala81 is conserved only in DDX43 orthologs but not in the other KH domains (Fig. S7, *E*–*F*).

To determine whether the length of the oligonucleotide had any effect on the binding affinity, we titrated the KH-89 protein with dT_10_ (Fig. S7*D*) and found that the *Kd* value was reduced 10-fold to 0.174 ± 0.090 mM, compared with 1.502 ± 0.290 mM for dT_5_ (Fig. 2*G*), suggesting that longer ssDNA or ssRNA substrates are preferred by the DDX43 KH domain. This also supports that much lower *Kds* were determined by filter-binding assays (Fig. S4), where longer substrates were used.

### Alanine-81 adjacent to the GRGG loop is critical for protein stability and nucleic acid binding

To understand the role of the conserved A81 in DDX43, we investigated the effect of side-chain size and polarity at this position by generating the A81G, A81I, and A81S mutant variants of KH-89. Like alanine, glycine and isoleucine are nonpolar amino acid residues, but, whereas glycine has no side chain at all, isoleucine has a branched aliphatic side chain, which is much bulkier than the methyl group of alanine. These differences might affect the KH-domain interaction with nucleotides, if alanine is important for proper positioning and interactions with nucleic acids. Similarly, the polar hydroxyl group in serine could interfere with the KH-domain interaction with nucleic acids. The A81G and A81S mutant proteins were soluble and successfully purified under the same conditions of the WT protein (Fig. S8, *A*–*B*). However, the A81I mutant was expressed poorly and could not be purified to homogeneity (Fig. S8, *C*–*D*).

**Figure 4:**
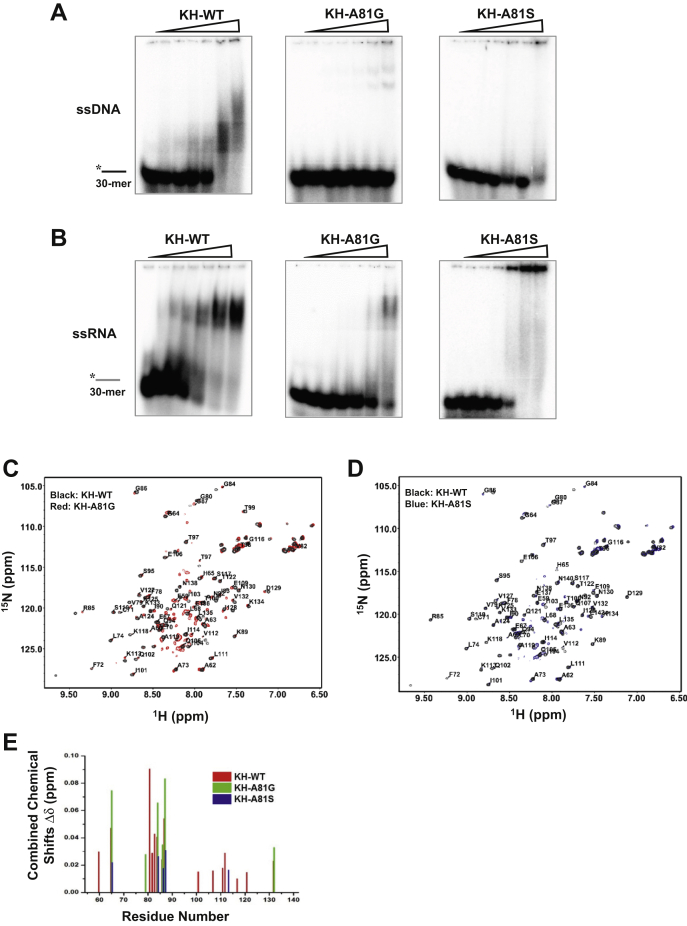
**The alanine adjacent to the GRGG loop is critical for nucleic acid binding.***A*–*B*, the representative EMSA images of increasing protein concentration (0–9.6 μM) of DDX43 KH domain (89 aa, including WT, A81G, and A81S) binding with 0.5 nM of ssDNA (A) or ssRNA (B). *C*–*D*, peak assignments of NMR spectra for KH-A81G (C) and A81S (D) and overlap of WT. *E*, combined chemical shift changes caused by dT_5_ binding to the KH-domain protein (89 aa, including WT, A81G, and A81S) as a function of the amino acid residue number. EMSA, electrophoretic mobility shift assay.

First, we performed EMSA and found that ssDNA and ssRNA binding was reduced significantly for the A81G and A81S mutants compared with the WT (Fig. 4, *A*–*B*). The ^1^H-^15^N HSQC NMR spectra of both mutant proteins were quite similar to those of the WT (Fig. 4, *C*–*D*), with the same positions of most peaks (Fig. 4*E*), indicating that mutant proteins are properly folded, similar to those of the WT. However, these mutations affected protein stability, causing faster precipitation of the proteins than that in the WT (data not shown). On titration of the mutant proteins with the dT_5_ oligonucleotide, we found that fewer amino acid residues showed chemical shift changes in A81G and A81S than the WT (Fig. 4, *C*–*E*). Interestingly, most residues involved in dT_5_ binding showed smaller chemical shift changes in A81S and larger chemical shift changes in A81G than the WT. Steric hindrances introduced by the hydroxyl group in serine might explain the lower binding affinity and smaller DNA-induced chemical shift changes than that in glycine, which is more similar to alanine in size. The stronger DNA-induced chemical shifts in the A81G mutant may be due to some repositioning of the oligonucleotide in the binding site, compared with the WT.

### SELEX and ChIP/CLIP-seq identification of nucleic acid sequences bound by the KH domain

**Figure 5:**
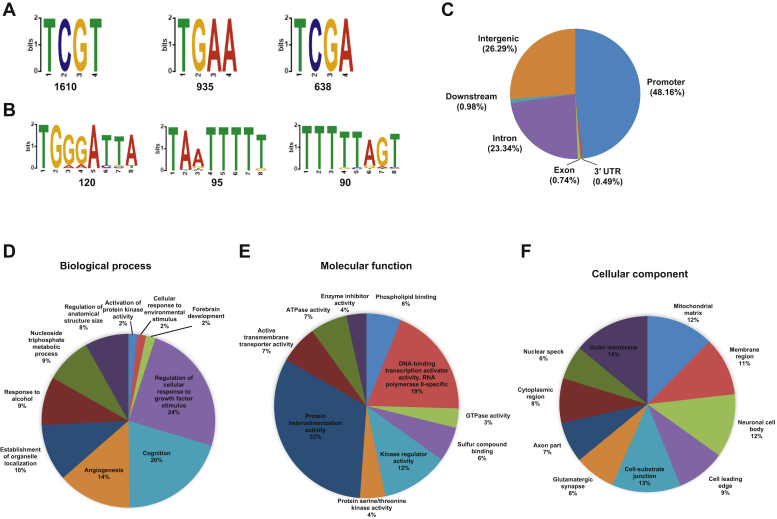
**Identification of DDX43 KH domain bound DNA by the SELEX and ChIP-seq methods.***A*, top three motif sequences obtained by the SELEX method. *B*, top three motif sequences obtained by the ChIP-Seq method. *C*, distribution of DDX43 KH-domain peaks across the human genome. *D*–*F*, distribution of gene ontology terms among the annotated sequences for biological process (*D*), molecular function (*E*), and cellular component (*F*). ChIP-seq, chromatin immunoprecipitation sequence; SELEX, systematic evolution of ligands by exponential enrichment.

Our EMSA and NMR results suggested that the DDX43 KH domain has a nucleic acid sequence binding preference. To identify the specific sequence(s), we first used a high-throughput systematic evolution of ligands by exponential enrichment (SELEX) approach. We chose a synthetic DNA library that contained ∼1 × 10^15^ of 20-mer random nucleotides (TriLink Biotechnologies). After subtracting reads from the control, we obtained 8878 of 20-nt sequences that bound to the KH-89 protein. We then searched these sequences for common motifs using the Multiple Expectation maximization for Motif Elicitation server (35). Because one single KH domain binds ∼4 nucleotides (8, 9), we initially selected a 3- to 5-nt window width. This search produced three sequences, with TCGT having the highest site count, 1610 of 8878 (Fig. 5*A*). A search with a 6- to 8-nt window width resulted in three top consensus sequences (Fig. S9*A*), two of which included the TCGT sequence. We also used the width ranging from 5 to 20 nt, and it resulted in two consensus sequences both containing the TCGT sequence (Fig. S9*B*).

To identify DNA bound by the KH domain *in vivo*, we used the chromatin immunoprecipitation (ChIP)-seq technique. After aligning with human hg38 and subtracting the vector control, we obtained 5.2 and 4.7 million reads with an average read length of 116 bp and 110 bp, corresponding to 67.5% and 67.3% mappable reads, respectively, for the duplicates. The MEME server search revealed three top conserved motifs with high T or G content (Fig. 5*B*). We mapped the reads to the human genome and found 48.16% of them to be located in the promoter regions, 26.29% in the intergenic regions, 23.34% in the introns, and a small fraction in the exons, 3′UTR, and downstream regions (Fig. 5*C*). Based on gene ontology (GO) analysis, the top three potential biological processes are regulation of cellular response to growth factor stimulus (24%), cognition (20%), and angiogenesis (14%); the top three potential molecular functions are protein heterodimerization activity (32%), DNA-binding transcription activator activity (19%), and kinase regulator activity (12%); and the top two potential cellular components are the outer membrane (14%) and cell-substrate junction (13%, Fig. 5, *D*–*F*). Similar results were obtained from another repeat (Fig. S10).

**Figure 6:**
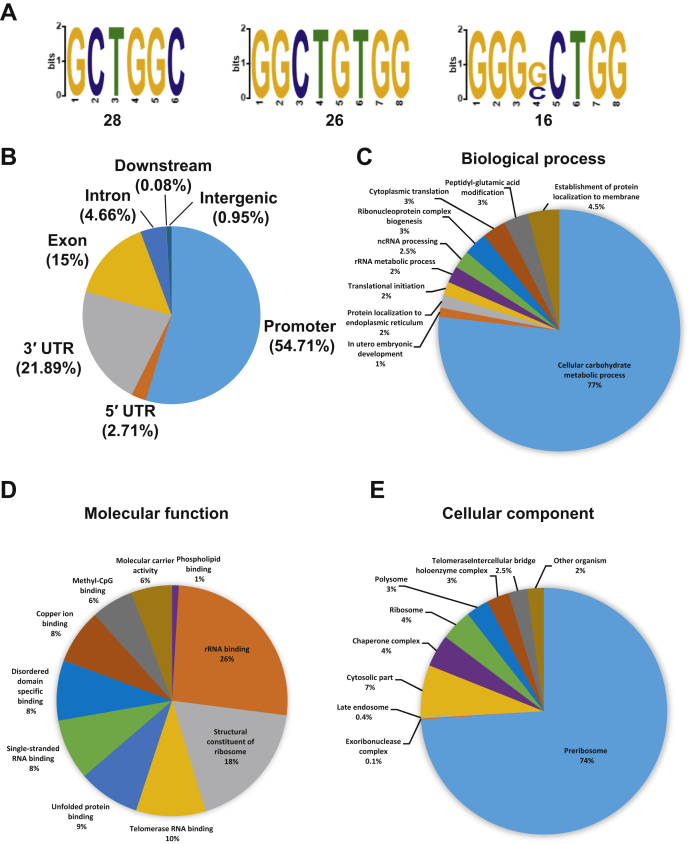
**Identification of DDX43 KH domain bound RNA by the CLIP-seq method.***A*, top three motif sequences obtained by the ChIP-Seq method. *B*, distribution of DDX43 KH-domain peaks across the human genome. *C*–*E*, distribution of gene ontology terms among the annotated sequences for biological process (*C*), molecular function (*D*), and cellular component (*E*). ChIP-seq, chromatin immunoprecipitation sequence; CLIP-seq, cross-linking immunoprecipitation sequence.

To identify RNA bound by the KH domain *in vivo*, we used the cross-linking immunoprecipitation (CLIP)-seq technique. For the RNA CLIP library duplicates, we obtained 5.5 and 5.7 million reads with an average sequence length of 60 bp and 65 bp, corresponding to 85.0% and 83.3% mappable reads, respectively. MEME analysis resulted in three top consensus sequences with CTG- or G-rich motifs (Fig. 6*A*). We mapped the reads to the human genome and found 54.71% of them located in the promoter regions, 21.89% in 3′UTR, and 15% in the exons and a small fraction in the introns, 5′UTR, and intergenic and downstream regions (Fig. 6*B*). The GO analysis revealed that the top potential biological process is cellular carbohydrate metabolic process (77%); the top three potential molecular functions are rRNA binding (26%), structural consistency of ribosome (18%), and telomerase RNA binding (10%); the top potential cellular component is the preribosome (74%, Fig. 6, *C*–*E*). Similar results were obtained from another repeat (Fig. S11).

### NMR validation of pyrimidine-rich sequences bound by the KH domain

**Figure 7:**
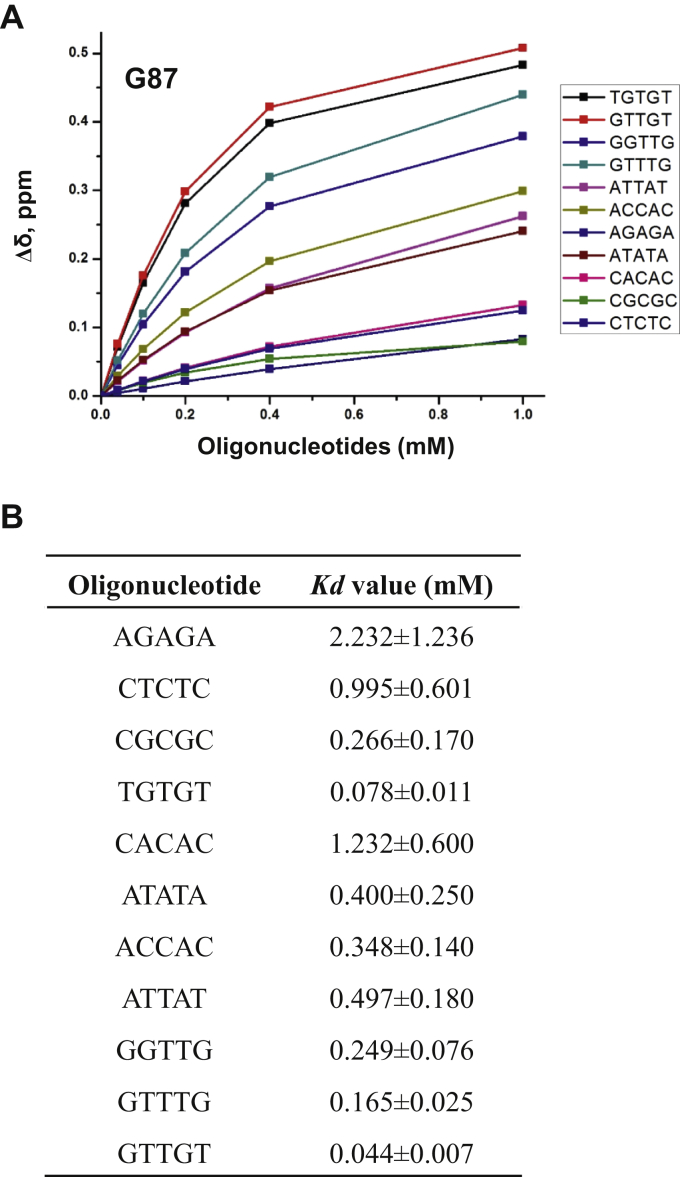
**Affinity of DDX43 KH domain with the oligonucleotide determined by NMR spectroscopy.***A*, combined chemical shift change (Δδ) plotted as a function of different oligonucleotide concentrations (0–1 mM) at 200 μM DDX43 KH-89 protein for residue G87. *B*, affinities of DDX43 KH domain (89 aa) with indicated oligonucleotides determined by NMR spectroscopy.

To identify and validate the sequences identified by SELEX and ChIP-/CLIP-seq, next we examined binding of different sequences to the KH-89 protein by ^1^H-15N HSQC NMR spectroscopy (Fig. S12). We calculated dissociation constants for various oligonucleotides from the chemical shift changes of G84 and G87, which are located in the nucleic acid binding loop and produce easily identifiable signals in an uncrowded region of the NMR spectrum. First, we examined chemical shift changes caused by homopurine sequence AGAGA and homopyrimidine sequence CTCTC and found that the DDX43 KH domain has a higher affinity for the pyrimidine sequence (Fig. 7, *A*–*B*), corroborating the findings from EMSA. Analyzing an array of purine-pyrimidine mixed sequences, we found that (1) TGTGT has the lowest *Kd* (0.078 mM) (2), adenine is not a preferred nucleotide as evidenced by high *Kd* values for ATATA, ACCAC, CACAC, and ATTAT (*Kd* ranging from 0.348 to 1.232), and (3) guanine increases binding affinity, especially adjacent to a pyrimidine such as in CGCGC and TGTGT. Among the pyrimidines, thymine is preferred over cytosine, as demonstrated by comparing binding affinities of TGTGT (*Kd* = 0.078 mM) and CGCGC (*Kd* = 0.266 mM) (Fig. 7*B*). This is consistent with a slightly higher binding affinity of dT_5_ than dC_5_ (Fig. 2*G*). Finally, we embedded guanines in various positions among thymines: GGTTG, GTTTG, and GTTGT. We found that GTTGT has the highest binding affinity (*Kd* = 0.044 mM), suggesting TTGT as a preferred motif for the DDX43 KH domain.

### The KH domain is crucial for the substrate specificity and unwinding processivity of the full-length DDX43 protein

**Figure 8:**
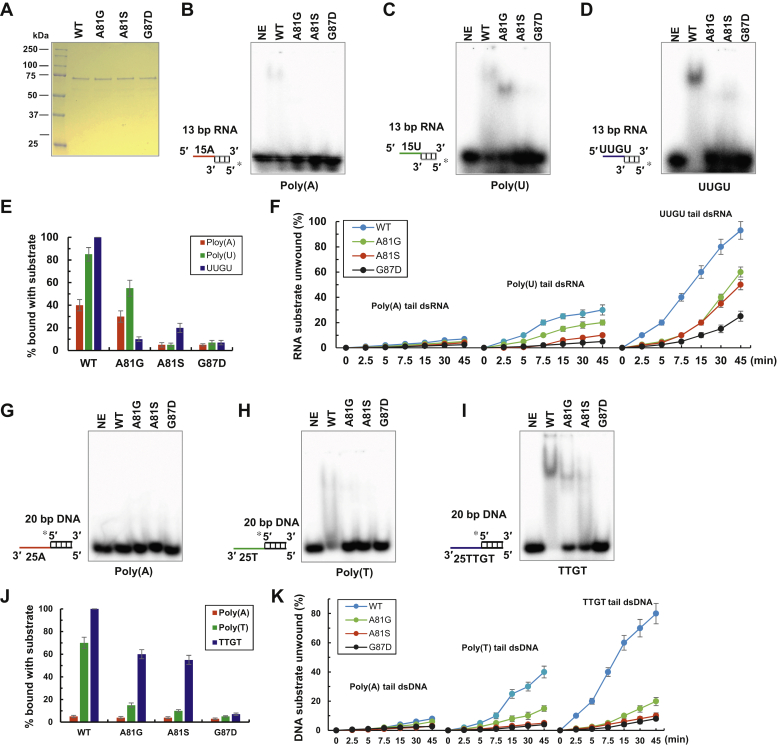
**Mutated alanine (A81) or glycine (G87) affects DDX41 in binding and unwinding processivity.***A*, the SDS-PAGE analysis of purified DDX43 full-length proteins (WT and mutants, 1 ug each). *B*–*E*, the representative images of EMSA performed by incubating DDX43 full-length proteins (2 μM) with 0.5 nM of 13-bp duplex RNA with 5′ tail of polyA (*B*), polyU (*C*), UUGU repeats (*D*), and their quantitative analysis (*E*). *F*, the quantitative analysis of helicase assays of DDX43 proteins (9.6 μM) on 0.5 nM of 13-bp duplex RNA with 5′ tail of polyA, polyU, or UUGU repeats as a function of time (0–45 min). *G*–*J*, the representative images of EMSA performed by incubating DDX43 full-length proteins (2 μM) with 0.5 nM of 20-bp duplex DNA with 3′ tail of polyA (*G*), polyT (*H*), TTGT repeats (*I*), and their quantitative analysis (*J*). *K*, the quantitative analysis of helicase assays of DDX43 proteins (9.6 μM) on 0.5 nM of 20-bp duplex DNA with 3′ tail of polyA, polyT, or TTGT repeats as a function of time (0–45 min). The *triangle* indicates heat-denatured RNA or DNA substrate control. Data are presented as the mean ± SD, n = 3. EMSA, electrophoretic mobility shift assay; NE, no enzyme.

The alanine (Ala81) adjacent to the GRGG loop and the last glycine (Gly87) in the GRGG loop are critical for nucleic acid binding of the DDX43 KH domain. To understand the roles of A81 and G87 in the context of the full-length DDX43, we introduced A81G, A81S, and G87D mutations into the full-length DDX43 and purified these proteins (Fig. 8*A*). DDX43 is capable of unwinding dsRNA regardless of the presence of a single-strand tail, but it prefers a 5′→3′ direction (30). Therefore, we designed 13-bp dsRNA substrates with a 5′ tail of 15-nt rU, rA, or rUUGU repeats (Table S2). First, we performed EMSA assays and found that DDX43-WT had a higher affinity than the mutants and that the substrate with rUUGU was preferred (Fig. 8, *B*–*E*). Next, we examined unwinding as a function of time (0–45 min) and found that DDX43-WT had a robust unwinding activity on the duplex RNA with a rUUGU overhang, less activity on the dsRNA with a poly(U) tail, and almost no activity on the dsRNA with a poly(A) tail (Fig. 8*F* and Fig. S13). For the preferred rUUGU tailed substrate, the unwinding rate is 2.17 ± 0.14 bp/min for WT, 0.87 ± 0.07 bp/min for both A81G and A81S, and 0.29 ± 0.03 bp/min for G87D. In general, the WT had a higher unwinding activity than the three mutants, with an order of WT>A81S(A81G)>G87D. Similarly, we tested DDX43-WT and the mutants on DNA substrates. Because DDX43 is a 3′→5′ DNA helicase (30), initially we designed a 20-bp dsDNA with a 15-nt 3′ tail, the same length as the tested RNA substrates; however, we could not detect any unwinding activity at the protein concentration used (2 μM). Then, we increased the 3′ tail length to 25 nt. EMSA revealed that all proteins preferred binding the TTGT substrate and that the WT had higher affinity than mutants (Fig. 8, *G*–*J*). Unwinding assays revealed that WT could unwind the dsDNA with 25-nt TTGT repeats or a poly T tail (lesser extent) but completely failed to unwind the dsDNA with a 25-nt poly A tail (Fig. 8*K* and Fig. S13). Again, WT protein had higher unwinding activity than mutants. For the preferred TTGT tailed substrate, the unwinding rate is 4.09 ± 0.33 bp/min for WT, 1.34 ± 0.12 bp/min for both A81G and A81S, and 0.47 ± 0.05 bp/min for G87D. Helicase assays as a function of protein revealed a similar conclusion: WT had higher unwinding activity than the A81G and G87D mutants (Fig. S14). Taken together, we concluded that the KH domain partially regulates the substrate specificity and unwinding processivity of the full-length DDX43 protein.

## Discussion

Although KH domains have been reported in many RNA-binding proteins (8, 9), the role of the KH domain in human helicases remains unknown. In fact, DDX43 and DDX53 are the only two helicases that contain a KH domain in their N-termini (Fig. S1). In this study, we addressed the binding specificity of the KH domain in DDX43 and its contribution to the helicase unwinding activity. Using EMSA, SELEX, ChIP-/CLIP-Seq, site-directed mutagenesis, and NMR, we found that the KH domain in the DDX43 helicase prefers to bind ssDNA and ssRNA, with a preference for pyrimidine-rich sequences, particularly the tetranucleotide TTGT. Furthermore, we showed that the KH domain is crucial for the unwinding processivity of the DDX43 helicase.

Isolated KH domains have been crystallized as monomers, dimers, and tetramers (8). We found that the DDX43 KH domain forms a monomer and a dimer and that the dimer has a higher affinity toward nucleic acids. We and others have reported that the full-length DDX43 protein exists as a monomer (30, 32); therefore, KH domain dimerization does not occur in the context of full-length protein. Crystal structure shows that the first KH domain of PCBP2 in complex with ssDNA forms two identical dimer complexes per asymmetric unit (11), but no dimer or higher order oligomers have been shown *in vivo*. We also purified full-length DDX43 protein in the presence of dT_8_ but did not obtain dimeric form (data not shown), indicating that the nucleic acid does not affect the oligomeric state of the DDX43 protein.

The conserved GXXG loop is a hallmark of the KH domains. This loop forms one wall of the bent oligonucleotide binding site and interacts with the phosphate backbone of the central nucleotides, forcing their bases into specific recognition pockets that determine base specificity. Both glycines are contacting the oligonucleotide and the presence of a side chain at these positions would introduce steric hindrance. Moreover, the C-terminal glycine assumes the backbone conformation that is attainable only for a glycine (positive φ/ψ torsion angles). Indeed, mutation of the first glycine in any of the two KH domains of the coding region determinant-binding protein (CRD-BP) protein completely abolishes the RNA binding activity *in vitro* (36). Similarly, replacing the first glycine, Gly227, with Asp or Ser in GXXG of GLD-1 results in a loss-of-function phenotype (37). We previously also showed that changing the first glycine, Gly84, to aspartic acid in DDX43 disrupts the binding to ssDNA/RNA substrates (30). In contrast, not much is known about the importance of the second glycine residue in the GXXG loop. It has been shown that the second glycine (G342) in KSRP KH3 forms hydrophobic bonds with the ribose of a guanine (position 4) in a RNA sequence AGGGU (38). Our EMSA data show that the Gly87 of the GRGG loop in the DDX43 KH domain is important for proper functioning of the full-length protein, and that mutation of Gly87 to aspartic acid reduces binding to the substrates (Fig. 8). In addition to the two conserved Gly residues, we discovered that an alanine adjacent to the GRGG loop is also crucial for nucleic acid binding, which is consistent with the notion that the five amino acid residues N-terminal to the GXXG motif discriminate between the first two bases of ssDNA or a ssRNA tetrad (39). Given that on average more than 15 amino acid residues in the KH domain contact nucleotides (8, 9, 39), our NMR results also demonstrated that more than 15 residues exhibit chemical shifts after nucleic acid binding (Fig. 3 and Fig. S7, *A*–*D*). Therefore, it is expected that other residues in the KH domain, besides Ala81, Gly84, and Gly 87, will contribute to the nucleotide binding and/or sequence specificity of the DDX43 protein.

Further investigation is required for the biological function of pyrimidine-rich sequences, especially TTGT, preferred by DDX43. Pyrimidine preference can be explained by the narrow width of the binding cleft in the KH domain of PCBP1, which would only accommodate the smaller pyrimidine bases but not the larger purines (40). It has been shown that the KH-containing protein PSI typically binds poly-pyrimidine RNA (41). Binding of hnRNP K and PCBP1 to the C-rich strand of human telomeric DNA was established *in vitro* (42) and in cell lines (43). Specific binding of hnRNP K to the single-stranded pyrimidine-rich sequence in the promoter of the human *c-Myc* gene activates transcription (44). Both our ChIP-seq and CLIP-seq data indicated that the DDX43 KH domain prefers to bind promoter regions (Figs. 5*C* and 6*B*). In fact, promoters are hotspots for many RNA-binding proteins (45), suggesting their roles in transcriptional regulation (46). Rather than homothymine, TTGT is preferred by the DDX43 KH domain. Consistent with our findings, recently another group also reported that DDX43 protein has the highest affinity to a TG repeat sequence (32). TGT core promoter motif is found to be conserved in human, *Drosophila*, and *Arabidopsis* (47). Interestingly, TTGT is the optimal binding sequence for the KH2+KH3 domains of the far upstream element (FUSE) binding protein (FBP) (48). Nevertheless, further studies are required to determine the mechanisms of DDX43 binding with promoters.

**Figure 9:**
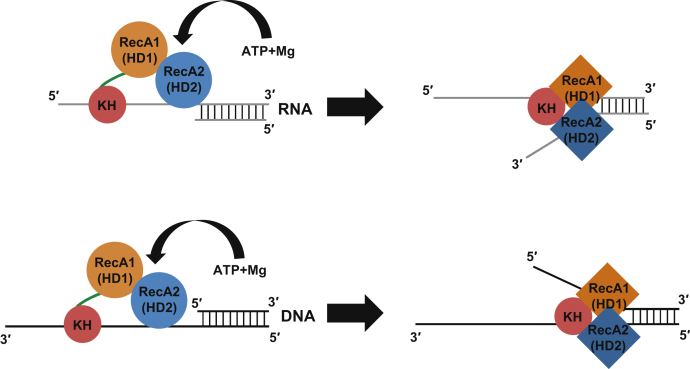
**Proposed models for the role of the KH domain in DDX43 helicase unwinding dsRNA (*upper*) and dsDNA (*bottom*).** In initial recognition and binding, the KH domain is mainly responsible for ssRNA/ssDNA binding. The helicase core domain, especially the RecA2 domain, might also be involved in nucleic acid binding. The binding of ATP and Mg causes conformational changes of two RecA domains. During unwinding, the KH domain binds with ssRNA/ssDNA (either strand) to stabilize RecA1/2 interacting with the fork junction and also prevent ssRNA/ssDNA reannealing. This, in turn, accelerates the unwinding processivity of helicase domains. RecA1 (HD1), RecA2 (HD2), and KH domains are shown in *orange*, *blue*, and *dark red*, respectively, and linkers are in *green*.

We have found that the KH domain in DDX43 is required not only for the full unwinding activity but also for substrate specificity. Our previous work showed that only the full-length DDX43 protein could unwind RNA and DNA duplex substrates and that the C-terminal helicase domain has no unwinding activity on RNA and very weak unwinding activity on DNA (30). This indicates that the KH domain at the N-terminus is crucial for DDX43 to perform its unwinding activity on substrates, which is supported by a recent finding demonstrating that the KH domain is more important than the helicase core domain for enzyme–substrate interaction (32). In the present work, we found that DDX43 could unwind, albeit less efficiently, nonpreferred RNA substrates (not containing UUGU tail) but not DNA substrates (Fig. 8). This might be due to DDX43’s ability to unwind dsRNA regardless of the single-stranded tail; however, it strictly requires a 3′ ssDNA tail on DNA substrates (30), where the KH domain initiates protein–nucleic acid binding. Canonical RNA helicases use a translocation-based duplex unwinding mechanism, where they first bind to a single-stranded region next to the duplex and then translocate in a unidirectional manner (49), whereas DEAD-box proteins load directly onto the duplex region and separate the two strands (50, 51). Our current data suggest that the KH domain may facilitate the DDX43 helicase to act like a canonical helicase. Three potential roles of the KH domain are as follows (1): to bind the single-stranded tail, that is, 5′ for RNA and 3′ for DNA, to initiate enzyme–substrate interaction; (2) to bind the single-stranded nucleotides, either pre-existing or newly separated, and stabilize RecA1/2 domains interacting with the fork junction; and (3) to bind separated single-stranded nucleotide chains to prevent them from re-annealing (Fig. 9). Many helicases require a separate ssDNA-binding protein for activity stimulation (52, 53, 54, 55), whereas DDX43 contains both functional domains within the same polypeptide.

DDX43 is overexpressed in a variety of cancers and thus can serve as a biomarker and a possible drug-target molecule. The expression of DDX43 is a potential prognostic marker and a predictor of response to anthracycline treatment in breast cancer (56, 57). DDX43 expression promotes melanoma tumor growth and progression (31). Frequent high expression of DDX43 was also found in CML and acute myeloid leukemia (29, 58). DDX43 is upregulated in decitabine-resistant K562 cells as well (59). Higher stage and metastasis progression correlate with higher DDX43 expression (60). Recently, it was found that arresting miR-186 and releasing lncRNA H19 by DDX43 facilitate tumorigenesis in CML (61). Therefore, DDX43 is a potential target for drug development. Our current biochemical evidence suggests that the KH domain is partially responsible for substrate specificity and processivity of DDX43. Thus, the KH domain itself can be a candidate epitope for the development of the peptide vaccines against tumors. On the other hand, its preferred target sequence TTGT/UUGU could potentially be used as an antagonist to inhibit the DDX43’s oncogenic function.

## Experimental procedures

### Plasmid DNA

Full-length DDX43-pET28a was described previously (30). Various DDX43 KH-domain fragments were PCR-amplified using primers listed in Table S3 and cloned into the *Nde*I and *Xho*I sites of the pET28a vector (Novagen). A fragment encoding amino acids 54 to 142 of the DDX43 KH domain (89 aa) was cloned into the *Hind*III and *Xho*I sites of a pcDNA3.0 vector (Invitrogen). Point mutations were generated with QuikChange Site-Directed Mutagenesis kit (Agilent Technologies) using primers listed in Table S3. All plasmids were verified by DNA sequencing.

### Recombinant protein

The plasmid pET28a-DDX43 KH domain was transformed into *E. coli* Rosetta (DE3) pLysS cells (Novagen). The Rosetta pLys cells were grown at 37 °C in the terrific broth medium containing 30 μg/ml kanamycin and 34 μg/ml of chloramphenicol until the A_600_ reached 0.6 and then induced by the addition of 0.5-mM IPTG overnight at 18 °C. The cells were harvested by centrifugation at 5000 rpm for 20 min at 4 °C. The periplasmic material was removed from the cells as described (62). Briefly, the cells were suspended in 5 ml/g of cell mass of the hypertonic buffer solution (50-mM Hepes, pH 7.4, 20% sucrose, 1-mM EDTA) and centrifuged at 4000 rpm for 30 min at 4 °C. The cells were re-suspended in 5 ml/g of cell mass of the hypotonic solution (5-mM MgSO_4_) and incubated for 10 min on ice. Cells were then pelleted by centrifugation at 4000 rpm for 10 min at 4 °C and stored at −80 °C until used. The cell suspension was lysed by sonication in buffer A (25-mM Tris, pH 8.0, 0.15 M NaCl, 100-μM Tween 20, and 10% glycerol) supplemented with 1-mM PMSF and protease inhibitor (Roche Applied Science) at 4 °C, with 5 short bursts of 10 s at intervals of 5 min. The cell debris and inclusion bodies were removed by centrifugation at 45,000*g* for 45 min at 4 °C. Recombinant His-tagged proteins were subjected to a two-step purification using Nickel Affinity beads (Sigma) and a Sephacryl S-100 high-resolution gel filtration column (GE Healthcare). The supernatant was applied to the Ni-NTA beads equilibrated with buffer A, washed with 10 column volumes of buffer B (25 mM Tris pH 8.0, 500 mM NaCl, 100 μM Tween 20 and 10% glycerol) containing 25-mM imidazole and eluted with 5 column volumes of buffer B containing 250-mM imidazole. The high-yield protein fractions, as confirmed by SDS-PAGE, were pooled and subjected to size-exclusion chromatography on a Sephacryl S-100 column (GE Healthcare) equilibrated with buffer A. The fractions were collected at a flow rate of 0.5 ml/min with the same buffer. The fractions containing pure protein at high yield, as determined by SDS-PAGE, were pooled and concentrated. The full-length DDX43 proteins (WT and mutants) were purified as described (30). The protein concentration was determined by the Bradford method using bovine serum albumin (BSA) as a standard.

### Nucleic acid substrates

PAGE-purified oligonucleotides used for RNA or DNA substrates were purchased from IDT and are listed in Table S2. The DNA substrates were prepared as described previously (30). Briefly, a single-stranded oligonucleotide was 5′-end–labeled with [γ-^32^P] ATP using T4 polynucleotide kinase (NEB) at 37 °C for 1 h. Unincorporated radionucleotides were removed by a G25 chromatography column (GE Healthcare). ssDNA or RNA substrates were kept at 4 °C and ready to use. For the ssDNA substrates, a [γ-^32^P]ATP-labeled oligonucleotide was annealed to a 2.5-fold excess of the unlabeled complementary strands in an annealing buffer (10 mM Tris HCl, pH 7.5, and 50 mM NaCl) by heating at 95 °C for 5 min and then cooling slowly to RT. For ssRNA, a [γ-^32^P]ATP-labeled oligonucleotide was annealed to a 2.5-fold excess of the unlabeled complementary strands in an annealing buffer (10-mM Mops, pH 6.5, 1-mM EDTA, and 50-mM KCl) by heating at 95 °C for 5 min and then cooling slowly to RT. All double-stranded substrates were purified by PAGE, and their concentrations were determined by liquid scintillation counting before use.

### Helicase assays

Helicase assay reaction mixtures (20 μl) contained 40-mM Tris (pH 8.0), 0.5-mM MgCl_2_, 15-mM NaCl, 0.01% Nonidet P-40, 0.1-mM DTT, 1 mg/ml BSA, equimolar mixture of 2-mM ATP and MgCl_2_, 0.5 nM of the specified duplex RNA or DNA substrate, and the indicated concentrations of DDX43 protein. Helicase reactions were initiated by the addition of DDX43 and then incubated at 37 °C for 15 min. Reactions were quenched with the addition of 20 μl of 2× stop buffer (17.5-mM EDTA, 0.3% SDS, 12.5% glycerol, 0.02% bromophenol blue, 0.02% xylene cyanol). For duplex RNA and DNA substrates, a 10-fold excess of unlabeled oligonucleotide (cold oligo) with the same sequence as the labeled strand was included in the quench to prevent reannealing. For kinetic assays, DDX43 protein and substrate were preincubated on ice, reactions were initiated by adding ATP, and 20-μl samples were quenched at the indicated times. The products of the helicase reactions for duplex RNA substrates were resolved on nondenaturing 15% (19:1 acrylamide: N, N'-methylenebisacrylamide) polyacrylamide gels, and products of DNA unwinding reactions were resolved on nondenaturing 12% (19:1 acrylamide: N, N'-methylenebisacrylamide) polyacrylamide gels. Radiolabeled DNA or RNA species in polyacrylamide gels were visualized using a PharosFX Imager and quantitated using the Quantity One software (Bio-Rad). The percent helicase substrate unwound was calculated by using the following formula: % unwinding = 100 × (P/(S + P)), where P is the product and S is the substrate. The values of P and S were corrected by subtracting background values in the no-enzyme and heat-denatured substrate controls, respectively. To estimate unwinding rate, we used the slowest protein (G87D) at the maximum time point (45 min) and then determined the time point when WT and A81G/S proteins reached the same unwinding percentage based on their linear fits to the time-course trajectories. The unwinding rate = the length of duplex/time.

### EMSA

Protein/DNA or RNA binding mixtures (20 μl) contained the indicated concentrations of DDX43 and 0.5 nM of the specified ^32^P-end–labeled DNA substrate in the same reaction buffer as used for helicase assays (see above) without ATP. The binding mixtures were incubated on ice for 30 min after the addition of DDX43 protein. After incubation, 3 μl of the loading dye (74% glycerol, 0.01% xylene cyanol, 0.01% bromphenol blue) was added to each mixture, and samples were loaded onto the native 5% (19:1 acrylamide/N, N'-methylenebisacrylamide) polyacrylamide gels and electrophoresed at 200 V for 2 h at 4 °C using 1× Tris/Borate/EDTA (TBE) as the running buffer. The resolved radiolabeled species were visualized using a PharosFX Imager (Bio-Rad).

### Filter-binding assay

The filter-binding assays were performed as described previously (63) with slight modifications. Briefly, the [^32^P]-radiolabeled RNA or DNA substrate (0.5 nM) was incubated with an increasing concentration of DDX43-KH-126 protein (0–9.6 μM) in a 50-μl total volume of binding buffer (25-mM Tris HCl, pH 7.5, 25-mM KOAC, 1 mM Mg(OAC)_2_, 1-mM DTT, 100 μg/ml BSA) at 37 °C for 30 min. The mixture was then filtered through a nitrocellulose membrane (0.45 μm, Millipore) using a dot-blot apparatus (Bio-Rad). After filtering, the wells were washed thrice with 100 μl of the wash buffer (25-mM Tris HCl, pH 7.5, 25-mM KOAC, 1-mM Mg(OAC)_2_) and the membranes were dried at RT for 10 min and quantified using PhosphorImager and Quantity One software (Bio-Rad). The *Kd* value was determined using GraphPad Prism software.

### NMR experiments

The DDX43-KH-89 protein was ^15^N labeled as described previously (64) and purified as described above. The NMR samples contained 0.2-mM DDX43-KH-89 protein, 0.25 M NaCl, 25-mM Hepes (pH 7.4), 5% (v/v) D_2_O, and 1-mM sodium 2,2-dimethyl-2-silapentane-5-sulfonate (chemical shift reference). For titrations, oligonucleotides were added at a molar ratio of 0, 0.2, 0.5, 1.0, 2.0, and 5.0 relative to the protein. The ^1^H-^15^N HSQC experiments were conducted on a 600-MHz Bruker NMR spectrometer. The data were processed using NMRFx Processor and analyzed using NMRViewJ (65). Combined chemical shift change Δδ was calculated as [(Δδ^2^_NH_ + Δδ^2^_N_/25)/2]^1/2^, where Δδ_NH_ and Δδ_N_ are the chemical shift changes of the amide proton and nitrogen, respectively. The structure of DDX43-KH-89 was predicted using the Phyre 2 server (33). The peaks in ^1^H-^15^N HSQC spectrum corresponding to the backbone amide groups were assigned to amino acid residues using SPARTA+ (34). Oligonucleotide dissociation constants were calculated from the combined chemical shift change as a function of the oligonucleotide concentration using fast-exchange regime model as described previously (66).

### SELEX

As elucidated in Figure 5*A*, a library containing ∼1 × 10^15^ oligonucleotides with a 20-nt random sequence in the middle, flanked by two 23-nt PCR primers, a forward primer (5′-TAGGGAAGAGAAGGACATATGAT-3′) and a reverse primer (5′-TCAAGTGGTCATGTACTAGTCAA-3′), was obtained from TriLink Biotechnologies (O-32001-20). The oligonucleotide library was PCR-amplified, heated, and kept on ice and then subjected to selection for binding with purified recombinant DDX43-KH-89 protein. Briefly, 2.4 μM of purified DDX43-KH-89 protein was incubated with 4.8 μM of oligonucleotides at 4 °C for 30 min on a rotary mixer using Ni-NTA affinity beads that were equilibrated with the 1 × helicase reaction buffer (HRB) binding buffer (40-mM Tris HCl (pH 8.0), 0.5-mM MgCl_2_, 0.01% NP-40, BSA (1 mg/ml), 15-mM NaCl). The beads were centrifuged at 500*g* for 5 min, and the supernatant was discarded. The beads were washed 5 times with 1×HRB binding buffer to remove any nonspecific binding by centrifuging at 500*g* for 5 min, and the supernatant was discarded. Oligonucleotides were eluted by adding the elution buffer (25-mM Tris HCl (pH 8.0), 500-mM NaCl, 10% glycerol, and 200-mM imidazole), incubating for 30 min in rotation at 4 °C, and centrifuging at 500*g* for 5 min, and the supernatant was collected. DNA was isolated by phenol:chloroform:isoamyl alcohol (25:24:1, Invitrogen), precipitated by adding an equal volume of 100% isopropanol and 5- to 50-μg glycogen, washed with 70% ethanol, and dissolved in sterile ultrapure water. The eluted DNA fragments were PCR-amplified and denatured at 92 to 95 °C to form ssDNA. The ssDNAs were then subjected to the next round of selection with DDX43-KH-89 protein, where half as much protein was used in each round compared with the previous round. Six rounds of binding, elution, amplification, and enrichment were performed. After the final round of selection, DNA was purified and used for a DNA library construction using a NEBNext ultra II DNA library prep kit (E7645, NEB). The quality of the library was checked using Bioanalyzer 2100 (Agilent). The multiplexed DNA samples were combined and analyzed in one lane of 125 cycles with paired-end 125-nt reads on an Illumina HiSeq 2500 system.

### ChIP-seq and CLIP-seq

As elucidated in Figure 5*E*, CLIP was performed by transfecting HEK293T cells with pcDNA3-DDX43-KH-89-3×FLAG construct, and overexpression was confirmed by the Western blotting using an anti-FLAG antibody (Fig. S15). The pcDNA3 empty vector (containing 3×FLAG only) served as a control. The overexpressed protein was cross-linked with chromatin or RNA using formaldehyde (37%, 40 μl/ml of media) for 15 min and quenched with 1.5 M glycine (60 μl/ml). The cells were washed with cold PBS, suspended in 1 ml of the swelling buffer (25-mM Hepes (pH 7.4), 1.5-mM MgCl_2_, 10-mM KCl, 0.1% NP-40, 1-mM DTT, 0.5-mM PMSF, and protease inhibitor cocktail (Sigma)), and centrifuged at 500*g* for 10 min. The pellet was then suspended in 1 ml of the sonication buffer (50-mM Hepes (pH 7.9), 140-mM NaCl, 1-mM EDTA, 1% Triton X-100, 0.1% Na-deoxycholate, 0.1% SDS, 0.5-mM PMSF, and protease inhibitor cocktail). The DNA was sheared by sonication of 20 cycles (30 s/cycle) with 50% duty cycle and 1-min intervals between each cycle, followed by centrifugation at 14,000 rpm for 15 min. The lysate containing nucleic acid bound to DDX43-KH-89 protein was added to M2-FLAG beads (A2220, Sigma) and incubated for 2 h at 4 °C. The supernatant was discarded by centrifuging at 3000 rpm for 8 min at 4 °C, followed by the addition of NTN 500-mM NaCl buffer (0.5% NP40, 50-mM Tris (pH 7.4), and 500-mM NaCl) to the slurry of protein M2-FLAG beads and further rotated for 15 min at 4 °C. The beads were washed after removing the supernatant with NTN 150-mM NaCl buffer (0.5% NP40, 50-mM Tris (pH 7.4), and 150-mM NaCl) and rotated at 4 °C for 15 min. The beads were suspended in BC 100 buffer (25-mM Tris (pH 7.4), 100-mM NaCl, 10% glycerol, 0.1% Tween 20), and the DDX43-KH-89 protein was eluted by adding 3×FLAG peptide and incubating at 65 °C for 1 h. The DNA was extracted with phenol–chloroform, precipitated with 2×volume of ethanol, washed with 70% ethanol, and dissolved in 50 μl of ddH_2_O. For the DNA library, 20 ng of ChIP-ed DNA was end-repaired using NEBNext Ultra II End Prep buffer and Enzyme mix, adaptor-ligated, cleaned with 0.9×AMPure beads, and size-selected using 0.4×beads. The size-selected DNA was then amplified using (index 3, 4, 5, 6, 7, and 8) NEBNext Multiplex Oligos for Illumina and universal primers (E7335, NEB). The products were cleaned using 0.9×AMPure beads.

For RNA extraction and purification, Trizol reagent (Invitrogen) was used to isolate the sheared RNA. The isolated RNA was eluted in nuclease-free water. The RNA library was prepared using the NEBNext Ultra II directional library prep kit (E7760, NEB) and DNA library by NEBNext ultra II DNA library prep kit (E7645, NEB) according to the manufacturer's instructions. Briefly, for RNA library preparation, the RNA extracted (1.5 μg) was converted into cDNA using random primers provided with the kit. The second strand was synthesized with NEBNext Second Strand Synthesis Reaction Buffer with dUTP and the enzyme mix. The products were purified using 1.8×volume of AMPure beads and the cDNA eluted with nuclease-free water. This cDNA was end-repaired, adaptor-ligated, cleaned up using 0.9×AMPure beads, and size-selected using 0.4×beads. The size-selected DNA was then amplified using six NEBNext Multiplex Oligos (index 9, 1, 11, 12, 30, and 37) and universal primer (E7335, NEB). The final products were cleaned using 0.9×AMPure beads.

The quality of the library was checked by a Bioanalyzer 2100 (Agilent). The multiplexed DNA samples were combined and analyzed in one lane of 125 cycles with paired-end 125-nt reads on an Illumina HiSeq 2500 system (NRC, Saskatoon).

### Bioinformatics analysis

For the SELEX data, the paired-end SELEX reads were trimmed using cutadapt (v1.18) (67) to remove primers and extract the DNA insert. The resulting trimmed reads were decomposed into k-mers for k = 5 to 20. The set of k-mers present in the control-read pool was subtracted from the set of k-mers present in the positive-read pool. These k-mers, termed exclusive k-mers, were then sorted by the number of times they appeared in the positive set. *p*-values were assigned to each k-mer using Fisher’s exact test. The resulting *p*-values were adjusted for multiple comparisons using the Benjamini–Hochberg procedure and a false-discovery rate of 5%. The resulting set of significantly enriched k-mers was submitted to MEME (35) for motif discovery.

For the ChIP- and CLIP-seq data, the adaptors of the paired-end FASTQ reads were trimmed using cutadapt (v1.18) (67) and then analyzed with FASTQC (v0.11.8) [http://www.bioinformatics.babraham.ac.uk/projects/fastqc/] to ensure adaptor removal and read quality. Quality trimming was elided because of sufficient read quality across the entire length of the read (>25 Phred score). The resulting trimmed reads were mapped to the hg38 human reference genome using Bowtie 2 (v2.3.5) (68). The Burrows–Wheeler Transform index for hg38 was obtained from the NCBI FTP server. With the resulting mapped reads, peaks were called using MACS2 (69) in paired-end read mode and a *p*-value threshold of 1 × 10^−5^, a threshold used in other studies (70). The control/test reads were submitted jointly to account for background noise. The peaks were written out in BED format, which specifies the location of each peak in the genome among other attributes. The DNA sequences located at each peak were extracted using the getfasta command from BEDTools (v2.28.0) (71) and the collection of sequences submitted to MEME (35) for motif discovery. GO analysis was performed by uploading the processed BED files into the usegalaxy.org server. In this server, under ChIP-seq, the ChIP-seeker for ChIP peak annotation and pie chart generation were selected. Gencode v34 annotation.gtf file was used a standard. For the biological process, molecular function, and cellular component, the annotated gene name and scores obtained from the usegalaxy.org server were uploaded to the webgestalt.org website. The pie chart was plotted based on the *p*-values of the genes with best 10 significance levels. SELEX-seq, ChIP-seq, and CLIP-seq data are deposited in the Gene Expression Omnibus under accession number GSE144647.

### Statistical analysis

All statistical analyses were performed in Microsoft Excel. Results are reported as the mean ± SD of at least three independent experiments. Comparisons were analyzed using unpaired Student’s t-test or one-way ANOVA test.

## Data availability

All data shown are available in the manuscript and the supplementary materials.

## Conflict of interest

The authors declare that they have no conflicts of interest with the contents of this article.
