# Supplementary material for: The KH domain facilitates the substrate specificity and unwinding processivity of DDX43 helicase
Source: J Biol Chem. 2020 Nov 23;296:100085. doi: 10.1074/jbc.RA120.015824 (PMC7949032; doi:10.1074/jbc.RA120.015824)
Supplement: Supplementary Figures and Tables [file mmc1.pdf]

Table S1. Nucleic acids bound by KH domain

KH domain	GXXG sequence	Specific nucleic acids bound by the KH domain (5′-3′)	References
Sam 68-KH	GPQG	UAAA	(1)
Vigilin (*X. laevis*)	Vigilin has 14 KH domains	(A)nCU and CU(A)n	(2)
Vigilin (*M. musculus*)	Vigilin has 14 KH domains	CHHC or CHYC (H = A/C/U and Y = C/U)	(3)
SF1-KH	GPRG	UACUAAC	(4,5)
QKI-KH	GPRG	YUAAY(Y = C/U)	(6)
		NACUAAY-N(1-20)-UAAY (Y = C/U)	(7)
hnRNP K-KH3	GKGG	TCCC	(8,9)
FBP-KH3	GRNG	TTTT	(8,9)
FBP-KH4	GKGG	ATTC	(8,9)
PCBP1-KH3	GRQG	CUCUCCUUUCUUUUUCUUCUUCCCUCCCUA	(10)
PCBP2-KH1	GKKG	C rich (CCCT or ACCC)	(11)
PCBP2-KH3	GRQG	CCCT	(12)
Nova-2-KH3	GKGG	UCAC	(13)
NusA: KH1–KH2	GMRG (KH1);<br>GRNG (KH2)	GAACUCAAUAG	(14)
KSRP-KH1	GRGG	UAUUUA	(15)
KSRP-KH2	GKGG	UAUUUA	(15)
KSRP-KH3	GRSG	UAUUUA	(15)
		AGGGU	(16-18)
KSRP-KH4	GRGG	UAUUUA	(15)

References:

1. Lin, Q., Taylor, S.J. and Shalloway, D. (1997) Specificity and determinants of Sam68 RNA binding. Implications for the biological function of K homology domains. *J. Biol. Chem*, **272**, 27274-27280.
2. Kanamori, H., Dodson, R.E. and Shapiro, D.J. (1998) In vitro genetic analysis of the RNA binding site of vigilin, a multi-KH-domain protein. *Mol Cell Biol*, **18**, 3991-4003.
3. Mobin, M.B., Gerstberger, S., Teupser, D., Campana, B., Charisse, K., Heim, M.H., Manoharan, M., Tuschl, T. and Stoffel, M. (2016) The RNA-binding protein vigilin regulates VLDL secretion through modulation of Apob mRNA translation. *Nature communications*, **7**, 12848.
4. Liu, Z., Luyten, I., Bottomley, M.J., Messias, A.C., Houngninou-Molango, S., Sprangers, R., Zanier, K., Kramer, A. and Sattler, M. (2001) Structural basis for recognition of the intron branch site RNA by splicing factor 1. *Science*, **294**, 1098-1102.
5. Berglund, J.A., Chua, K., Abovich, N., Reed, R. and Rosbash, M. (1997) The splicing factor BBP interacts specifically with the pre-mRNA branchpoint sequence UACUAAC. *Cell*, **89**, 781-787.
6. Teplova, M., Hafner, M., Teplov, D., Essig, K., Tuschl, T. and Patel, D.J. (2013) Structure-function studies of STAR family Quaking proteins bound to their in vivo RNA target sites. *Genes Dev*, **27**, 928-940.
7. Galarneau, A. and Richard, S. (2005) Target RNA motif and target mRNAs of the Quaking STAR protein. *Nature structural & molecular biology*, **12**, 691-698.
8. Braddock, D.T., Baber, J.L., Levens, D. and Clore, G.M. (2002) Molecular basis of sequence-specific single-stranded DNA recognition by KH domains: solution structure of a complex between hnRNP K KH3 and single-stranded DNA. *Embo j*, **21**, 3476-3485.
9. Braddock, D.T., Louis, J.M., Baber, J.L., Levens, D. and Clore, G.M. (2002) Structure and dynamics of KH domains from FBP bound to single-stranded DNA.

Nature, **415**, 1051-1056.

10. Sidiqi, M., Wilce, J.A., Vivian, J.P., Porter, C.J., Barker, A., Leedman, P.J. and Wilce, M.C. (2005) Structure and RNA binding of the third KH domain of poly(C)-binding protein 1. *Nucleic Acids Res*, **33**, 1213-1221.
11. Du, Z., Lee, J.K., Fenn, S., Tjhen, R., Stroud, R.M. and James, T.L. (2007) X-ray crystallographic and NMR studies of protein-protein and protein-nucleic acid interactions involving the KH domains from human poly(C)-binding protein-2. *Rna*, **13**, 1043-1051.
12. Fenn, S., Du, Z., Lee, J.K., Tjhen, R., Stroud, R.M. and James, T.L. (2007) Crystal structure of the third KH domain of human poly(C)-binding protein-2 in complex with a C-rich strand of human telomeric DNA at 1.6 A resolution. *Nucleic Acids Res*, **35**, 2651-2660.
13. Lewis, H.A., Musunuru, K., Jensen, K.B., Edo, C., Chen, H., Darnell, R.B. and Burley, S.K. (2000) Sequence-specific RNA binding by a Nova KH domain: implications for paraneoplastic disease and the fragile X syndrome. *Cell*, **100**, 323-332.
14. Beuth, B., Pennell, S., Arnvig, K.B., Martin, S.R. and Taylor, I.A. (2005) Structure of a Mycobacterium tuberculosis NusA-RNA complex. *Embo j*, **24**, 3576-3587.
15. Hollingworth, D., Candel, A.M., Nicastro, G., Martin, S.R., Briata, P., Gherzi, R. and Ramos, A. (2012) KH domains with impaired nucleic acid binding as a tool for functional analysis. *Nucleic Acids Res*, **40**, 6873-6886.
16. Trabucchi, M., Briata, P., Garcia-Mayoral, M., Haase, A.D., Filipowicz, W., Ramos, A., Gherzi, R. and Rosenfeld, M.G. (2009) The RNA-binding protein KSRP promotes the biogenesis of a subset of microRNAs. *Nature*, **459**, 1010-1014.
17. Garcia-Mayoral, M.F., Diaz-Moreno, I., Hollingworth, D. and Ramos, A. (2008) The sequence selectivity of KSRP explains its flexibility in the recognition of the RNA targets. *Nucleic Acids Res*, **36**, 5290-5296.
18. Nicastro, G., Garcia-Mayoral, M.F., Hollingworth, D., Kelly, G., Martin, S.R., Briata, P., Gherzi, R. and Ramos, A. (2012) Noncanonical G recognition mediates KSRP regulation of let-7 biogenesis. *Nature structural & molecular biology*, **19**, 1282-1286.

Table S2. DNA and RNA substrates and oligonucleotides used in this study

Substrate name	Structure or description	Nucleotide sequence (5′→3′)
ssDNA (30 mer)	Used as random sequence ssDNA in EMSA	DNA 30 mer: GAGCTACCAGCTACCCCGTATGTCAGAGAG
Fork dsDNA (30 bp)	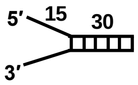	Fork 30/15-T:<br>TTTTTTTTTTTTTTTGGTGATGGTGTATTGAGT<br>GGGATGCATGCA<br>Fork 30/15-B:<br>TGCATGCATCCCACTCAATACACCATCACCTT<br>TTTTTTTTTTTTT
Blunt-end dsDNA (30 bp)	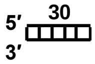	DNA 30 mer: GAGCTACCAGCTACCCCGTATGTCAGAGAG<br>DNA 30 mer comp:<br>CTCTCTGACATACGGGGTAGCTGGTAGCTC
ssRNA (30 mer)	Used as random sequence ssRNA in EMSA	RNA-30-mer:<br>GAGCUACCAGCUACCCCGUAUGUCAGAGAG
30-bp blunt-end dsRNA	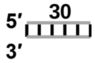	RNA-30-mer:<br>GAGCUACCAGCUACCCCGUAUGUCAGAGAG<br>RNA-30mer-comp:<br>CUCUCUGACAUACGGGGUAGCUGGUAGCUC
Forked dsRNA	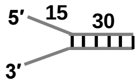	Fork RNA-30/15-T:<br>UUUUUUUUUUUUUUUGGUGAUGGUGUAUUGAGU<br>GGGAUGCAUGCA<br>Fork RNA 30/15-B:<br>UGCAUGCAUCCCACUCAAUACACCAUCACCUU<br>UUUUUUUUUUUUU
13-bp dsRNA with poly (A) 5′ tail	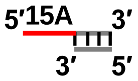	RNA-13 B(bottom strand): GCGUCUUUACGGU<br>RNA-41B_15A (top strand):<br>AAAAAAAAAAAAAAACCGUAAAGACGC
13-bp dsRNA with poly (U) 5′ tail	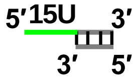	RNA-13 B(bottom strand): GCGUCUUUACGGU<br>RNA-41B_15U (top strand):<br>UUUUUUUUUUUUUUUACCGUAAAGACGC
13-bp dsRNA with UUGU repeats 5′ tail	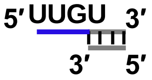	RNA-13 B(bottom strand): GCGUCUUUACGGU<br>RNA-28B_15UUGU (top strand):<br>UGUUGUUGUUGUUGUACCGUAAAGACGC
20-bp dsDNA with poly (A) 3′ tail	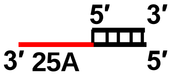	Fork 20/15-2 no T (top strand):<br>GGTGATGGTGTATTGAGTGG<br>Fork 20/25-1 A (bottom strand):<br>CCACTCAATACACCATCACCAAAAAAAAAAAAAAAAA<br>AAAAAAAA
20-bp dsDNA with poly (T) 3′ tail	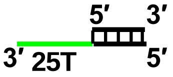	Fork 20/15-2 no T (top strand):<br>GGTGATGGTGTATTGAGTGG<br>Fork 20/25-1 T (bottom strand):<br>CCACTCAATACACCATCACCTTTTTTTTTTTTTTTTTTTTT<br>TTTT
20-bp dsDNA with TTGT repeats 3′ tail	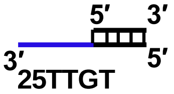	Fork 20/15-2 no T (top strand):<br>GGTGATGGTGTATTGAGTGG<br>Fork 20/25-1 TTGT (bottom strand):<br>CCACTCAATACACCATCACCTTGTTGTTGTTGTTGTTGT<br>TGTTGT
dT_8	Co-purified with DDX43 full-length protein	TTTTTTTT
dT_{10}	Used as ssDNA in NMR	TTTTTTTTTT

dT_{30}	Used as ssDNA in EMSA	TTTTTTTTTTTTTTTTTTTTTTTTTTTTTT
dA_{30}	Used as ssDNA in EMSA	AAAAAAAAAAAAAAAAAAAAAAAAAAAAAA
dC_{30}	Used as ssDNA in EMSA	CCCCCCCCCCCCCCCCCCCCCCCCCCCCCC
rU_{30}	Used as ssRNA in EMSA	UUUUUUUUUUUUUUUUUUUUUUUUUUUUUU
dT_5	Used as ssDNA in NMR	TTTTT
dA_5	Used as ssDNA in NMR	AAAAA
dC_5	Used as ssDNA in NMR	CCCCC
dG_5	Used as ssDNA in NMR	GGGGG
rU_5	Used as ssRNA in NMR	UUUUU
MY_CTCTC	Used as ssDNA in NMR	CTCTC
MY_CACAC	Used as ssDNA in NMR	CACAC
MY_ATATA	Used as ssDNA in NMR	ATATA
MY_CGCGC	Used as ssDNA in NMR	CGCGC
MY_ACCAC	Used as ssDNA in NMR	ACCAC
MY_ATTAT	Used as ssDNA in NMR	ATTAT
MY_GTTGT	Used as ssDNA in NMR	GTTGT
MY_AGAGA	Used as ssDNA in NMR	AGAGA
MY_TGTGT	Used as ssDNA in NMR	TGTGT
MY_GGTTG	Used as ssDNA in NMR	GGTTG
MY_GTTTG	Used as ssDNA in NMR	GTTTG

Table S3. Primers used in this study

Primer	Sequence (5'-3')	Used in this study
DDX43-KH-F-NdeI (74 aa)	ACGTCATATGCCGCTGTGTTTTGCTTTGAAG	Forward primer to PCR amplify DDX43 KH domain (74 aa) for cloning in *Nde*I site of pET28a vector
DDX43-KH-R-XhoI (74 aa)	GCATCTCGAGTTCTGAATTGTAATTTTCTTC	Reverse primer to PCR amplify DDX43 gene KH domain (74 aa) for cloning in *Xho*I site of pET28a vector
DDX43-KH126aa-Nde1-F	AGTCCATATGAGAGGTGGTCGCTGGAGAGGC	Forward primer to PCR amplify DDX43 KH-126 aa for cloning in *Nde*I site of pET28a vector
DDX43-KH126aa-Xho1-R	ACTGCTCGAGTCAATCTATCAATGGCCGATC	Reverse primer to PCR amplify DDX43 gene KH 126 for cloning in *Xho*I site of pET28a vector
DDX43-KH89aa-Nde1-F	ATATCATATGACCTCTAGGCCCCCGGAGGCC	Forward primer to PCR amplify DDX43 KH-89 aa for cloning in *Nde*I site of pET28a vector
DDX43-KH89-Xho1-R	CATGCTCGAGTCATTCTGAATTGTAATTTTC	Reverse primer to PCR amplify DDX43 gene KH 89 aa for cloning in *Xho*I site of pET28a vector
DDX43-KH80aa-Xho1-R	ATAGACAATTTTGTTAAATGACTCGAGCATG	Reverse primer to PCR amplify DDX43 KH 80 aa fragment for cloning in *Xho*I site of pET28a vector (using with primer DDX43-KH89aa-*Nde*1-F)
3xFLAG-F	AGCTTATGGACTACAAAGACCATGACGGTGATTATAAAGATCATGACATCGATTACAAGGATGACGATGACAAGTGAG	3xFLAG coding sequence. Annealed with 3XFLAG-R and cloned into *Hind*III and *EcoR*I site of pcDNA3.0 vector
3XFLAG-R	AATTCTCACTTGTCATCGTCATCCTTGTAATCGATGTCATGATCTTTATAATCACCGTCATGGTCTTTGTAGTCCATA	3xFLAG coding sequence. Annealed with 3XFLAG-F and cloned into *Hind*III and *EcoR*I site of pcDNA3.0 vector
pcDNA3-DDX43-KH89-Hind3-F	ACTGAAGCTTGCCGCCACCATGACCTCTAGGCCCCCGGAG	Forward primer to PCR amplify DDX43 KH-89 aa fragment for cloning in *Hind*III site of pcDNA3.0 vector
pcDNA3-FLAG-DDX43-KH89-Xho1-R	ACTACTCGAGTCACTTGTCATCGTCATCCTTGTAATCGATGTCATGATCTTTATAATCACCGTCATGGTCTTTGTAGTCTTCTGAATTGTAATTTTCTTC	Reverse primer with to PCR amplify DDX43 KH 89 fragment for cloning in *Xho*I site of pcDNA3.0 vector with a 3xFLAG tag
DDX43-A81I-F-new	GAAGAGCCACTTTGTTGGCATCGTAATCGGTCGTGGTGGGTC	Forward primer for site-directed mutagenesis of DDX43-KH-A81I
DDX43-A81I-R-new	GACCCACCACGACCGATTACGATGCCAACAAAGTGGCTCTTC	Reverse primer for site-directed mutagenesis of DDX43- KH-A81I
DDX43-A81G-F	GAAGAGCCACTTTGTTGGCGGGGTAATCGGTCGTGGTGGGTC	Forward primer for site-directed mutagenesis of DDX43-KH-A81G
DDX43-A81G-R	GACCCACCACGACCGATTACCCCGCCAACAAAGTGGCTCTTC	Reverse primer for site-directed mutagenesis of DDX43- KH-A81G
DDX43-A81S-F	GAAGAGCCACTTTGTTGGCTCGGTAATCGGTCGTGGTGGGTC	Forward primer for site-directed mutagenesis of DDX43-KH-A81S
DDX43-A81S-R	GACCCACCACGACCGATTACCGAGCCAACAAAGTGGCTCTTC	Reverse primer for site-directed mutagenesis of DDX43- KH-A8SI
DDX43-G87D-F	GTAATCGGTCGTGGTGACTCAAAAATAAAGAAT	Forward primer for site-directed mutagenesis of DDX43-G154D
DDX43-G87D-R	ATTCTTTATTTTTGAGTCACCACGACCGATTAC	Reverse primer for site-directed mutagenesis of DDX43-G154D
MY-Forward selection primer	TAGGGAAGAGAAGGACATATGAT	Forward primer to PCR amplify SELEX library insert (20 nt random sequence)
MY-Reverse selection primer	TCAAGTGGTCATGTACTAGTCAA	Reverse primer to PCR amplify SELEX library insert (20 nt random sequence)

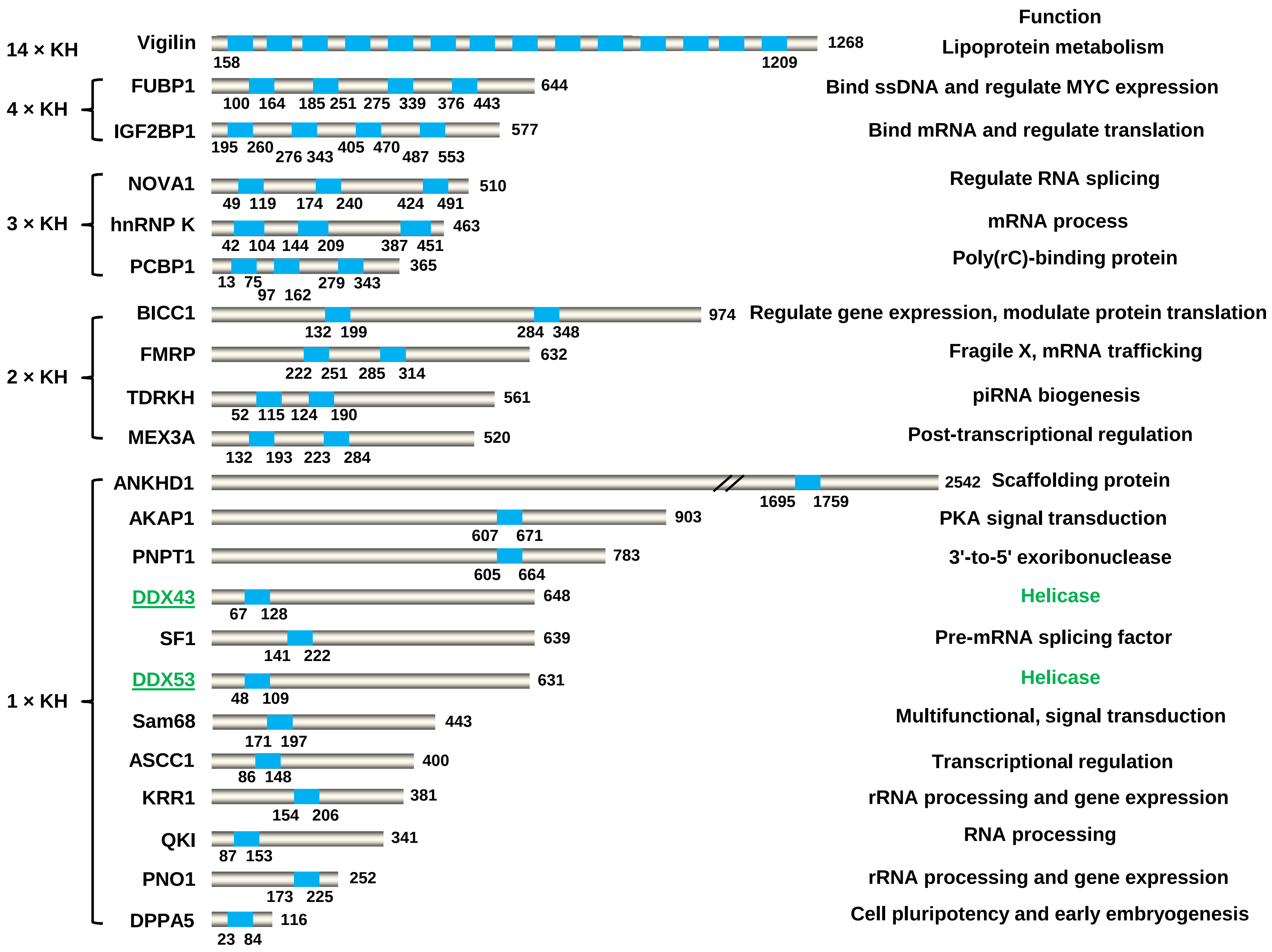

Function
14 × KH
Vigilin
158
1209
1268
Lipoprotein metabolism
4 × KH
FUBP1
100 164 185 251 275 339 376 443
644
Bind ssDNA and regulate MYC expression
IGF2BP1
195 260 276 343 405 470 487 553
577
Bind mRNA and regulate translation
3 × KH
NOVA1
49 119 174 240 424 491
510
Regulate RNA splicing
hnRNP K
42 104 144 209 387 451
463
mRNA process
PCBP1
13 75 97 162 279 343
365
Poly(rC)-binding protein
2 × KH
BICC1
132 199 284 348
974
Regulate gene expression, modulate protein translation
FMRP
222 251 285 314
632
Fragile X, mRNA trafficking
TDRKH
52 115 124 190
561
piRNA biogenesis
MEX3A
132 193 223 284
520
Post-transcriptional regulation
1 × KH
ANKHD1
1695 1759
2542
Scaffolding protein
AKAP1
607 671
903
PKA signal transduction
PNPT1
605 664
783
3'-to-5' exoribonuclease
DDX43
67 128
648
Helicase
SF1
141 222
639
Pre-mRNA splicing factor
DDX53
48 109
631
Helicase
Sam68
171 197
443
Multifunctional, signal transduction
ASCC1
86 148
400
Transcriptional regulation
KRR1
154 206
381
rRNA processing and gene expression
QKI
87 153
341
RNA processing
PNO1
173 225
252
rRNA processing and gene expression
DPPA5
23 84
116
Cell pluripotency and early embryogenesis

Figure S1. Summary of KH domain containing human proteins. A total of 40 human proteins contain KH domain(s). The KH domain region and protein function are adapted from Uniprot (www.uniprot.org/) and GeneCards (www.genecards.org) respectively. All proteins can bind RNA, which is not shown in the function. The DDX43 and DDX53 are highlighted in green. For the paralogs such as FUBP1 and FUBP3; FXR1, FXR2, and FMRP; MEX3A, MEX3B, MEX3C, and MEX3D; KHDRBS1, KHDRBS2, and KHDRBS3; PCBP1, PCBP2, PCBP3, and PCBP4; IGF2BP1, IGF2BP2, and IGF2BP3; NOVA1 and NOVA2, only one of them is shown.

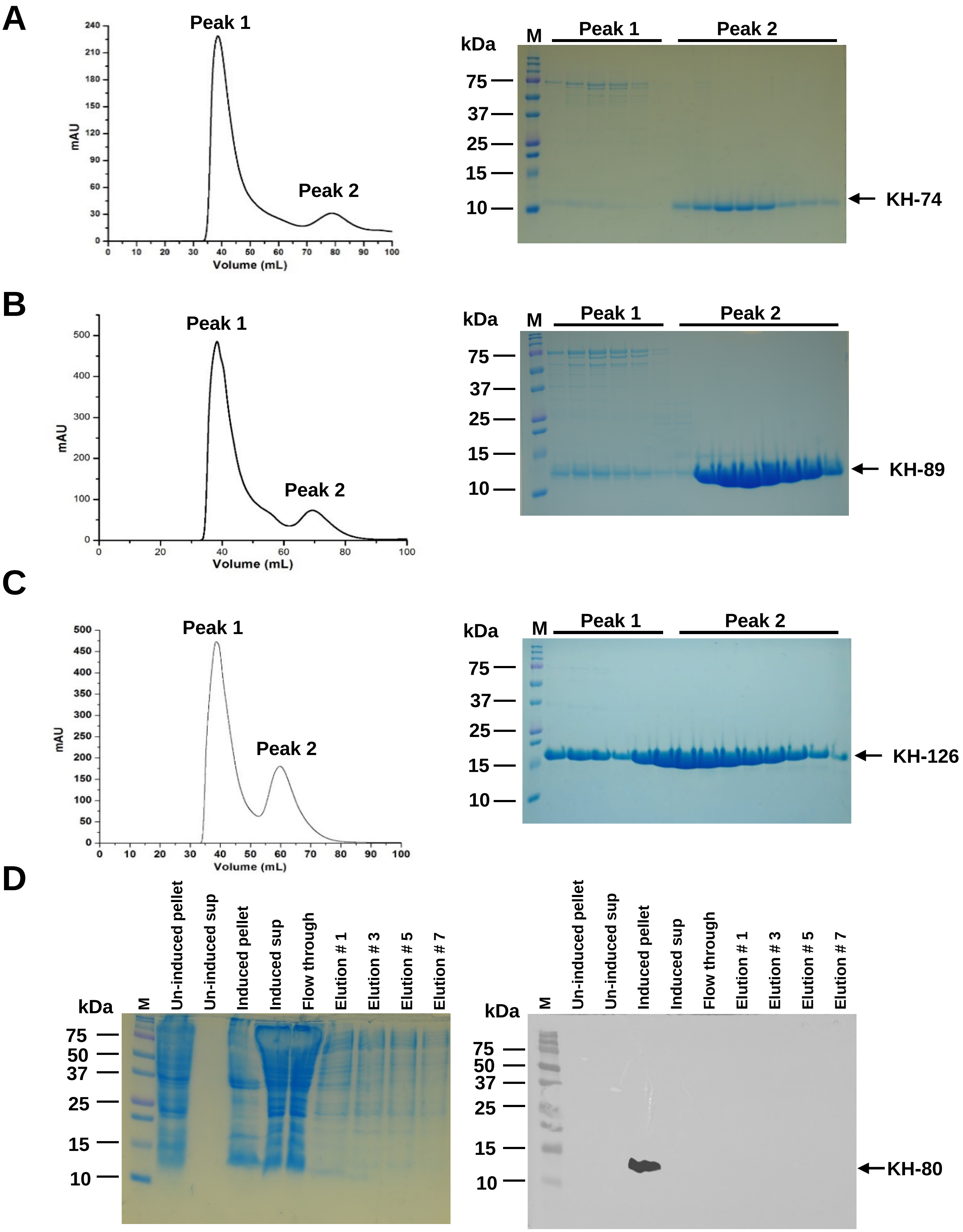

Figure S2. Purification of KH domain proteins. (**A-C**) Chromatographic profiles of recombinant KH domain proteins eluting from a Sephacryl S-100 column (left) and SDS-PAGE analysis of the eluted fractions (right). M, marker. (**D**) SDS-PAGE analysis of the expression and purification of DDX43-KH-80 protein (left) and Western blotting using an anti-His antibody (right).

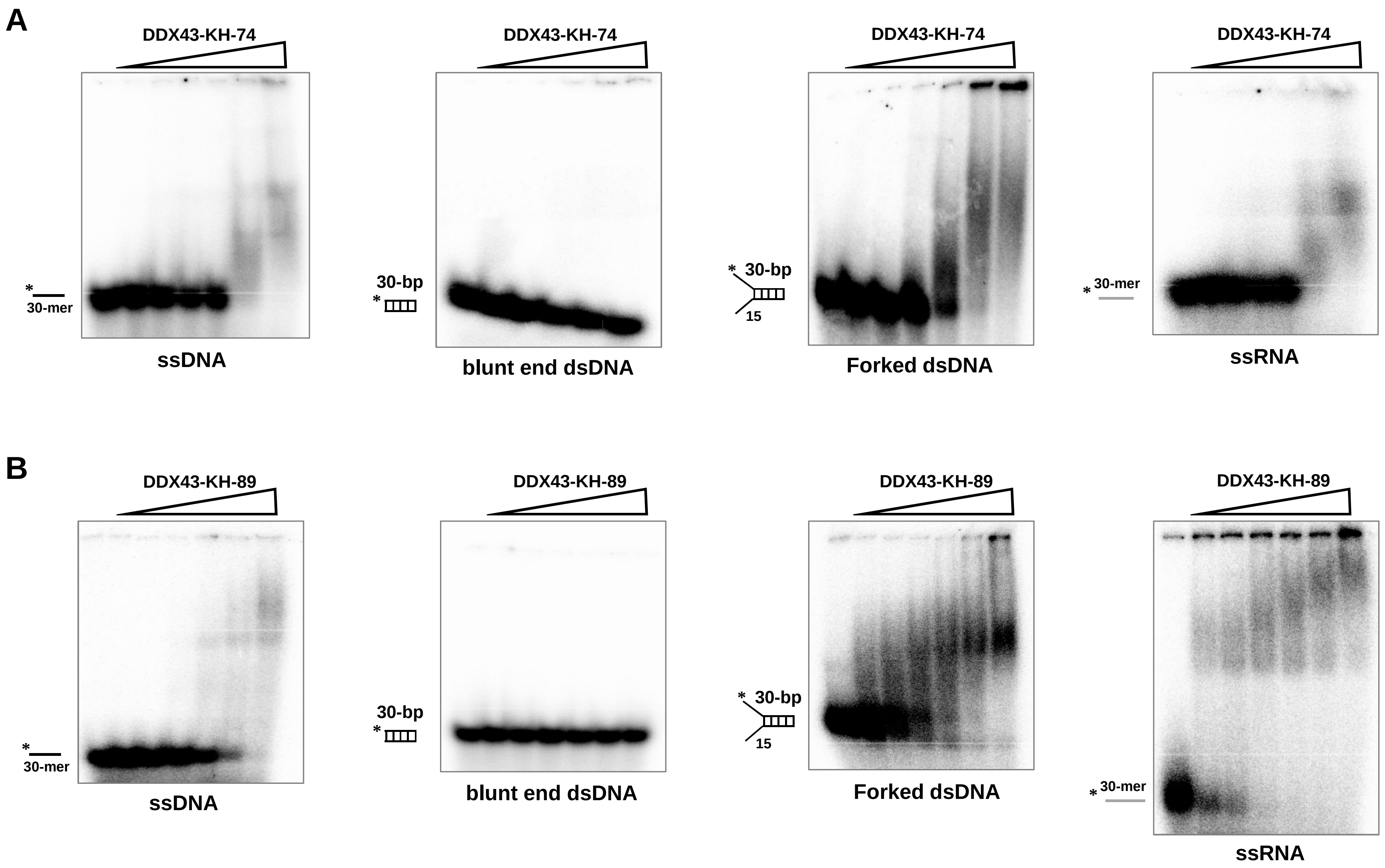

Figure S3. EMSA of DDX43 KH-74 and -89 proteins binding with different substrates. Representative EMSA images of increasing protein concentration (0–9.6 µM) of DDX43 KH domain proteins, 74aa (**A**) and 89aa (**B**), binding with 0.5 nM of indicated substrates. DNA is in black, RNA in gray.

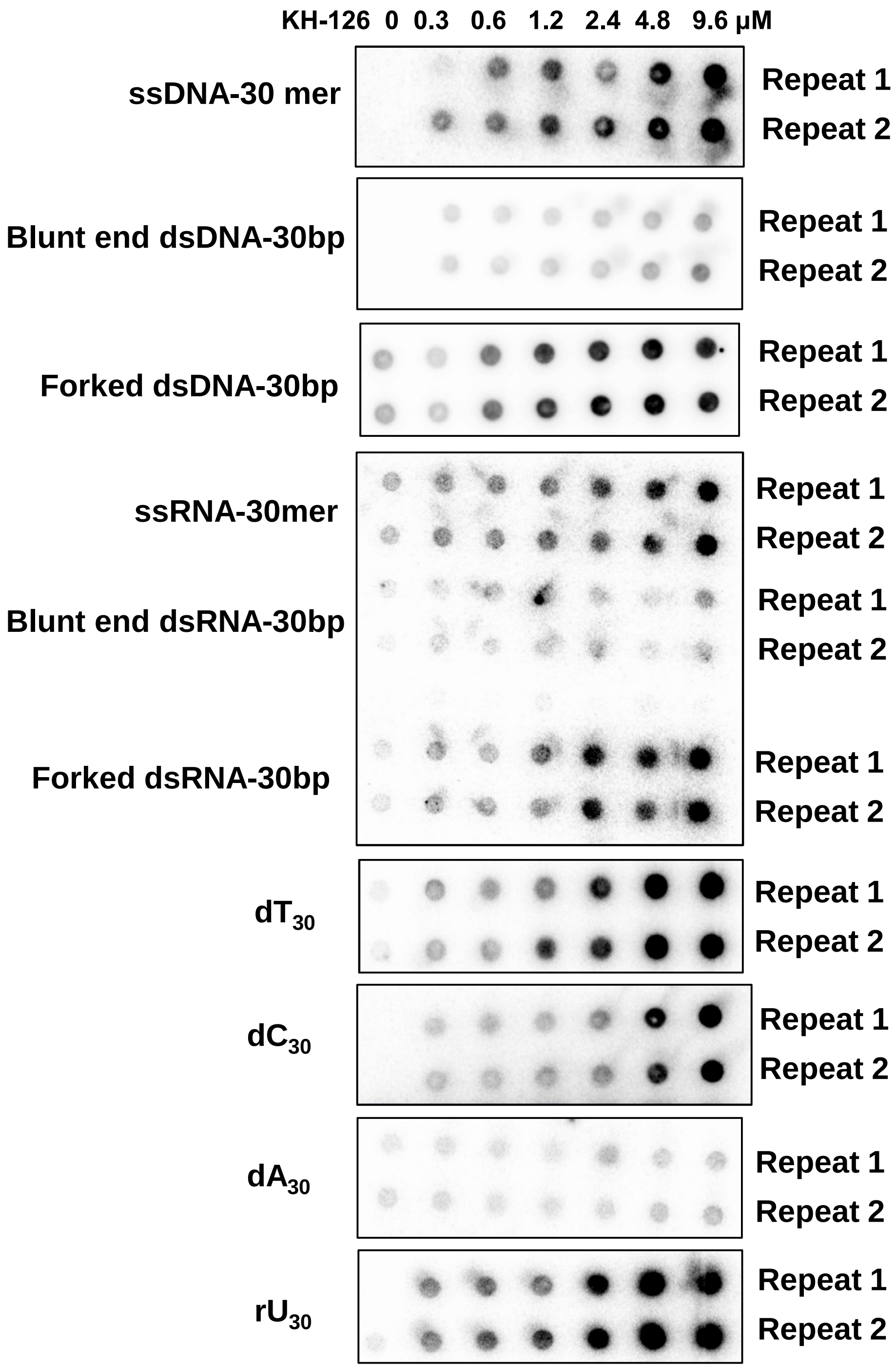

Figure S4. Representative images of filter binding assays. ^{32}P-labeled oligo or substrate (0.5 nM) was incubated with DDX43-KH 126 protein (0, 0.3, 0.6, 1.2, 2.4, 4.8, 9.6 µM) at 37°C for 30 min, then passed through a nitrocellulose membrane. After washing, bound radioactivity was visualized using a PharosFX Imager. Each sample has two repeats.

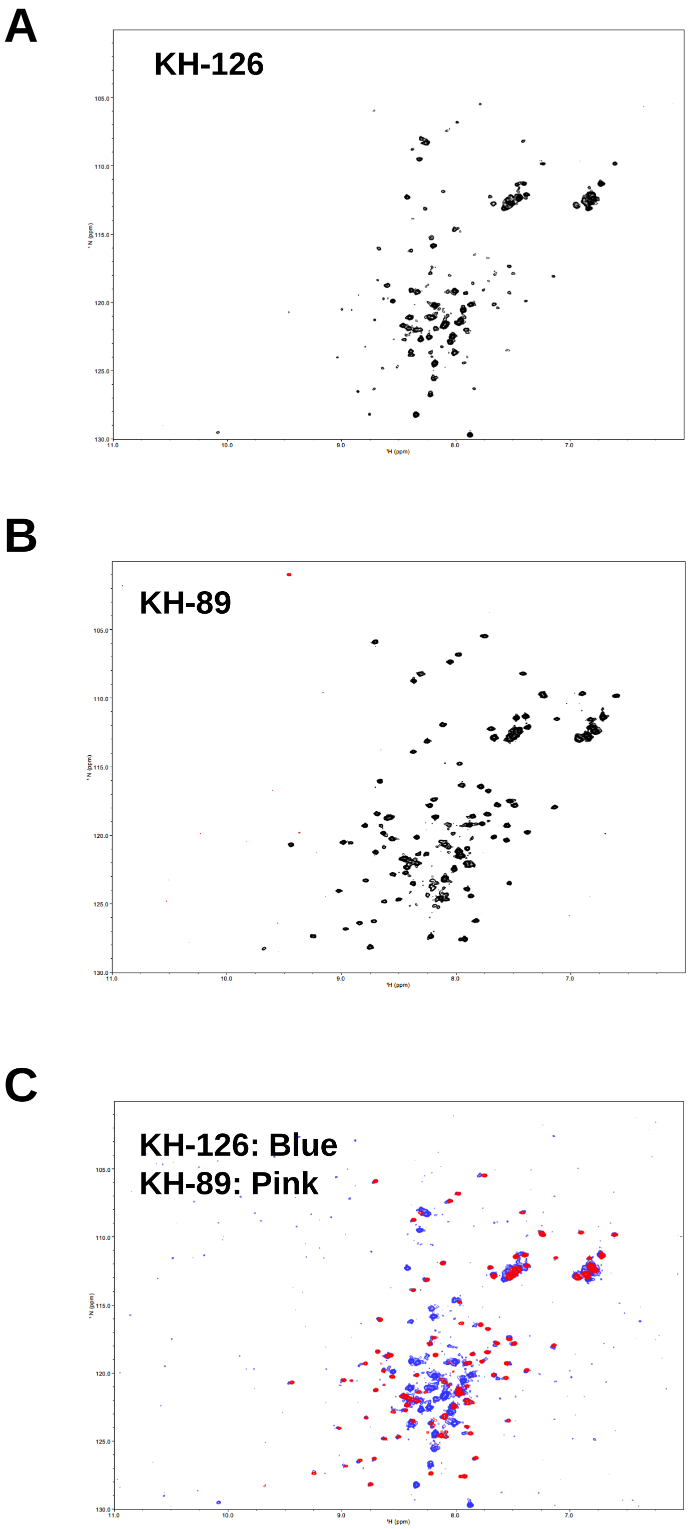

Figure S5. ^{1}H-^{15}N HSQC spectra of apo KH-126 and -89 proteins that have His tag. (**A and B**) ^{1}H-^{15}N HSQC spectra of KH-126 protein (A) and KH-89 protein (B). (**C**) Overlay of ^{1}H-^{15}N HSQC spectra of the KH-126 (blue) and KH-89 (pink) proteins.

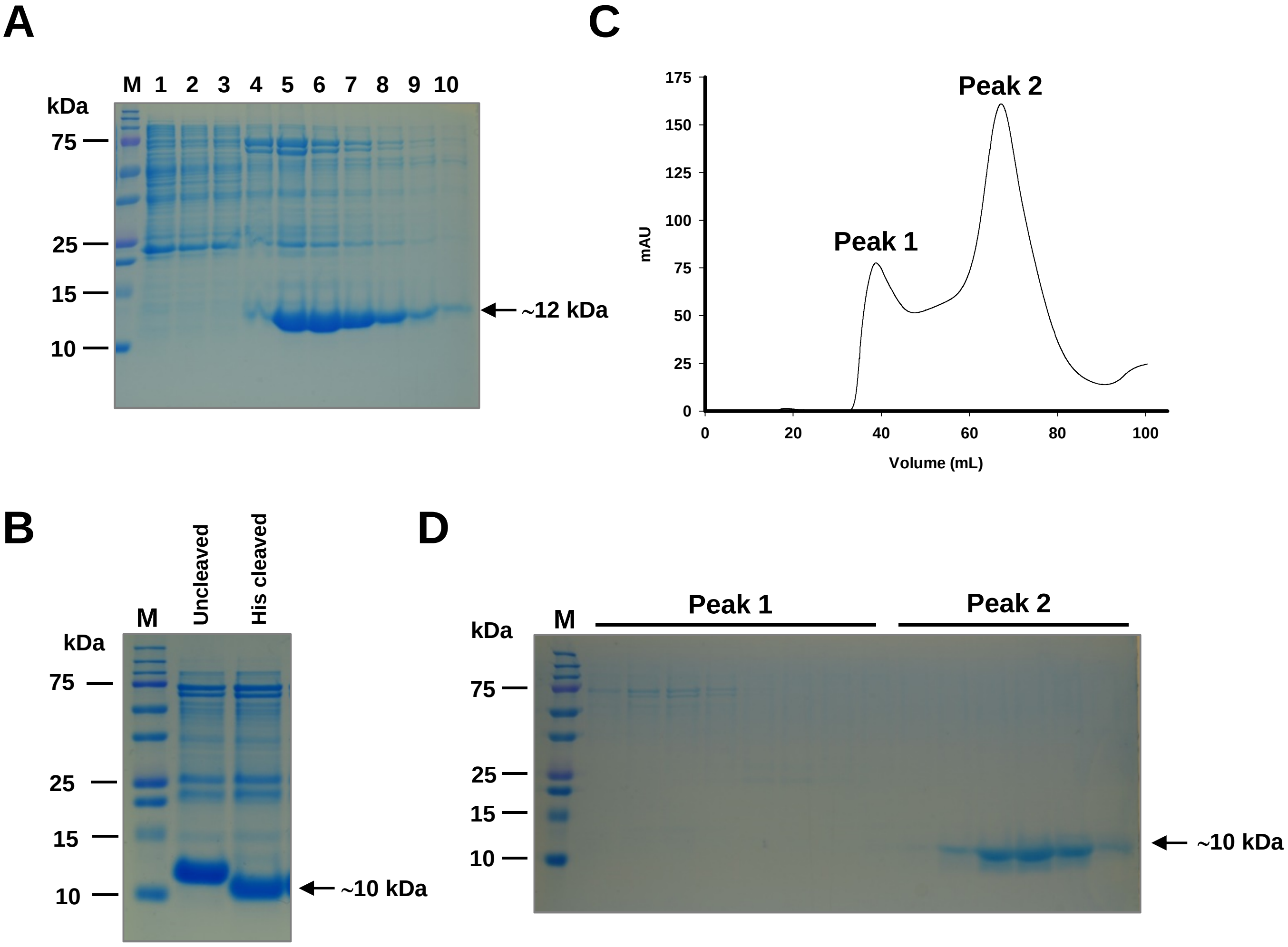

Figure S6. Purification of His tag cleaved DDX43 KH-89 protein for NMR analysis. (**A**) SDS-PAGE analysis of the eluted KH-89 fractions from a Ni-NTA affinity column by imidazole**.** M, marker; 1-10, fractions. (**B**) SDS-PAGE analysis of thrombin-cleaved and uncleaved KH-89 proteins. (**C**) Chromatographic profile of thrombin-cleaved KH-89 protein eluting from a Sephacryl S-100 column. (**D**) SDS-PAGE analysis of the KH-89 protein peaks eluted from the gel filtration chromatography in panel C.

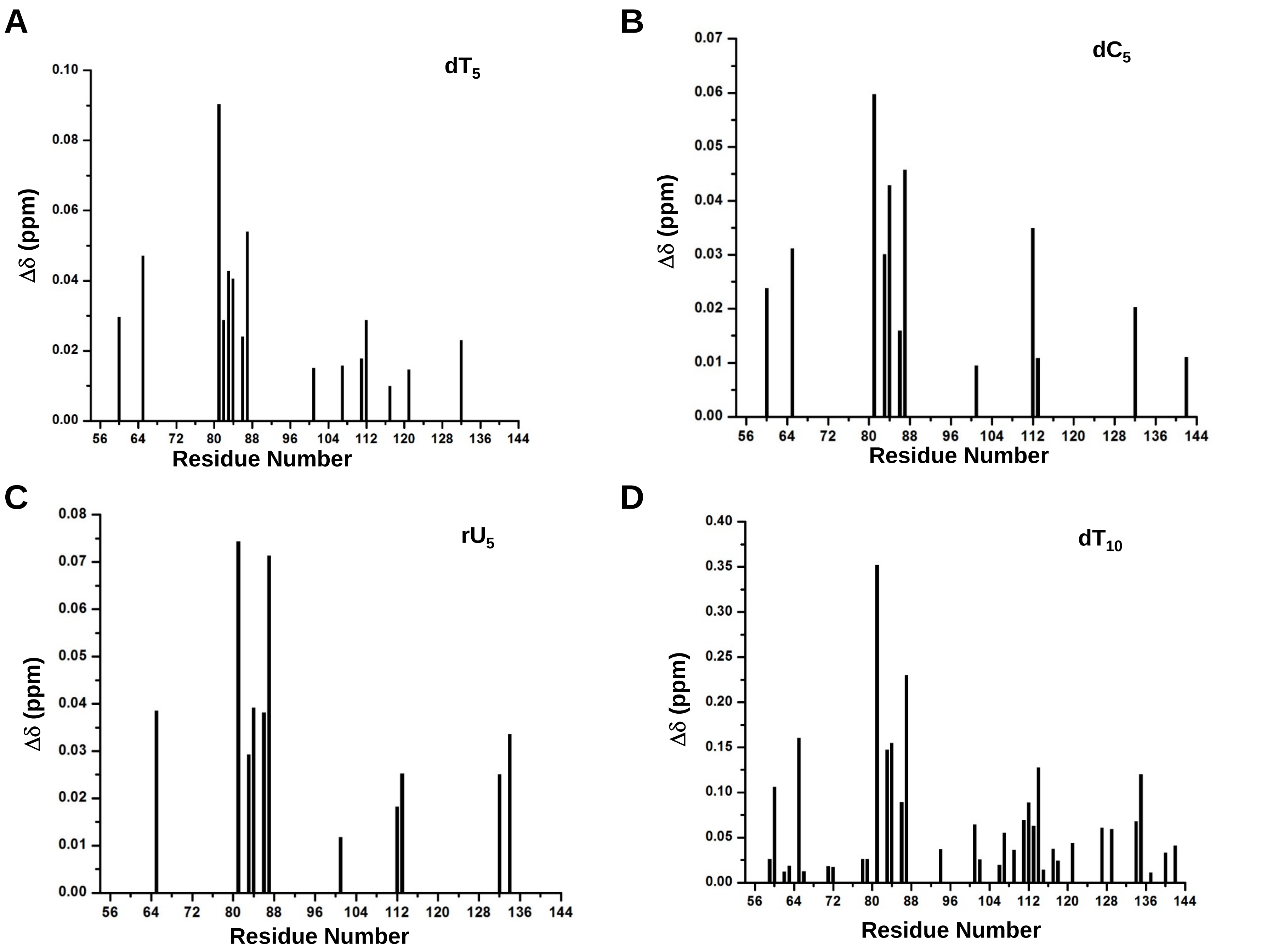
A
dT5
Δδ (ppm)
0.00
0.02
0.04
0.06
0.08
0.10
56 64 72 80 88 96 104 112 120 128 136 144
Residue Number
B
dC5
Δδ (ppm)
0.00
0.01
0.02
0.03
0.04
0.05
0.06
0.07
56 64 72 80 88 96 104 112 120 128 136 144
Residue Number
C
rU5
Δδ (ppm)
0.00
0.01
0.02
0.03
0.04
0.05
0.06
0.07
0.08
56 64 72 80 88 96 104 112 120 128 136 144
Residue Number
D
dT10
Δδ (ppm)
0.00
0.05
0.10
0.15
0.20
0.25
0.30
0.35
0.40
56 64 72 80 88 96 104 112 120 128 136 144
Residue Number

Figure S7

E

	GXXG
Homo sapiens	VGAVIGRGGSKIK
Macaca mulatta	VGAVIGRGGSKIK
Pan troglodytes	VGAVIGRGGSKIK
Rattus norvegicus	VGAVIGRGGSKIR
Mus musculus	VGAVIGRGGSKIR
Felis catus	VGAVIGRGGSKIK
Bos Taurus	VGAVIGRGGSNIK
Ovis aries	VGAVIGRGGSNIK
Anolis caroli nensis	VGALIGRGGSRIK
Piliocolobus tephrosceles	VGAVIGRGGSKIK
Microtus ochrogaster	VGAVIGRGGSKIR
Urocitellus parryii	VGAVIGRGGSKIK
Bos indicus x Bos Taurus	VGAVIGRGGSNIK
Lagenorhynchus obliquidens	VGAVIGRGGSKIK
Acinonyx jubatus	VGAVIGRGGSKIK
Zalophus californianus	VGAVIGRGGSKIK
Ursus arctos horribilis	VGAVIGRGGSKIK
	A81 G87

F

		A81 G87
hnRNP K	(1)	AGAVIGKGGKNIKAL
hnRNP K	(2)	AGGIIGVKGAKIKEL
hnRNP K	(3)	AGSIIGKGGQRIKQI
FMR-1	(1)	MGLAIGTHGANIQQA
FMR-1	(2)	VGKVIGKNGKLIQEI
NOVA	(1)	AGSIIGKGGQTIVQL
NOVA	(2)	AGLIIGKGGATVKAV
NOVA	(3)	VGAILGKGGKTLVEY
SF1		VGLLIGPRGNTLKNI
**DDX43**		VGAVIGRGGSKIKNI
KHDR1		VGKILGPQGNTIKRL
DDX53		VGVVIGYSGSKIKDL
TDRKH		VGRIIGRGGETIRSI
KSRP		VGVVIGRSGEMIKKI
PCBP2		CGSLIGKGGCKIKEI
AKAP149		VGRLIGKQGRYVSFL
ANKHD1		VSRIMGRGGCNITAI
RS3		TQNVLGEKGRRIREL
FUBP1		VGLIIGRGGEQINKI
IF2B3		AGRVIGKGGKTVNEL
M3XC		VGLVVGPKGATIKRI
VIGILIN		HKFLIGKGGGKIRKV
ANKRD17		ISRVIGRGGCNINAI
		GXXG

Figure S7. Nucleic acid bind to the same amino acids in the KH domain, especially A81 and G87. (A-D) Combined chemical shift change (Δδ) as a function of KH-89 (200 µM) residue number observed for dT_5 (A), dC_5 (B), rU_5 (C) and dT_{10} (D) oligonucleotide (400 µM) binding. (**E**) Amino acids A81 and G87 are conserved in DDX43 proteins from different eukaryotes. (**F**) Alignment of the GXXG motifs from DDX43 and other known human KH domains, showing G87 is conserved, but not A81.

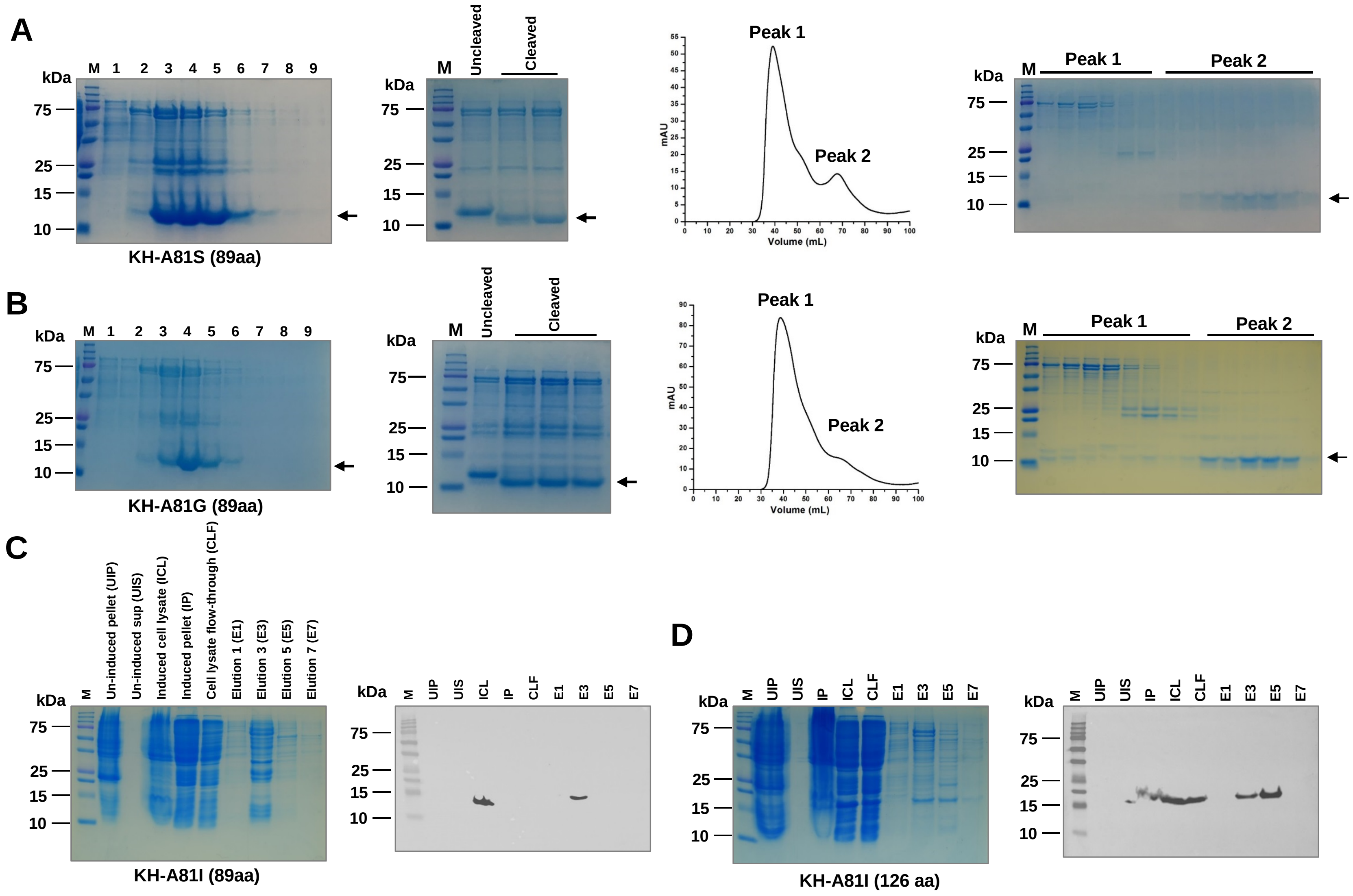

Figure S8. Purification of DDX43 KH-A81S, -A81G, and -A81I proteins. (**A and B**) SDS-PAGE analysis of the expression and purification of KH-A81S (A) and -A81G (B), and gel filtration profiles. His tag was cleaved by thrombin. M, marker. (**C and D**) SDS-PAGE analysis of the expression and purification of the KH-A81I protein (left) and Western blotting using an anti-His antibody (right) in KH-89aa (C) and KH-126aa (D) constructs.

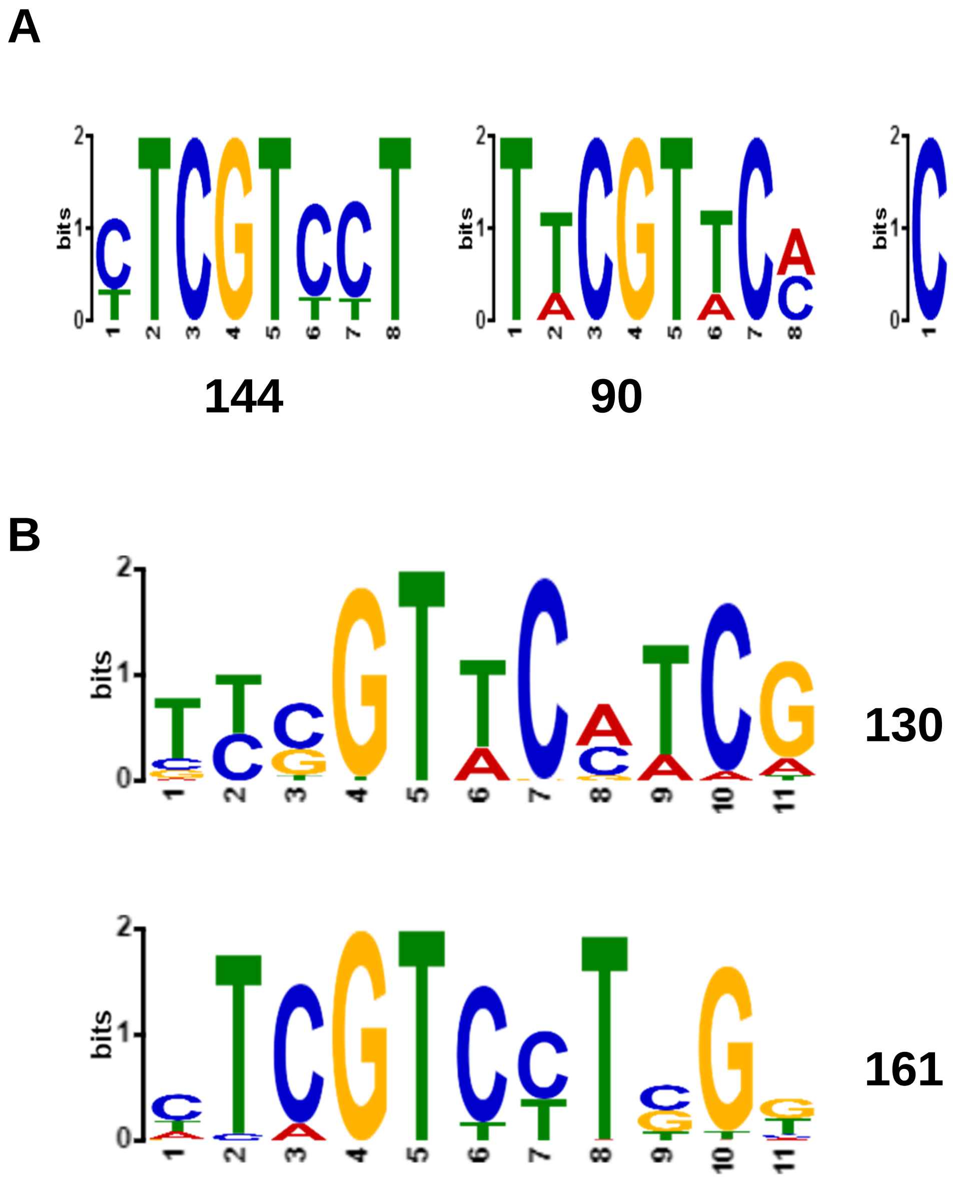

Figure S9. Top DNA sequence motifs identified by SELEX and analyzed by the MEME server with 6-8 nt (A) and 5-20 nt width (B). Number of sequences containing the respective motif are indicated.

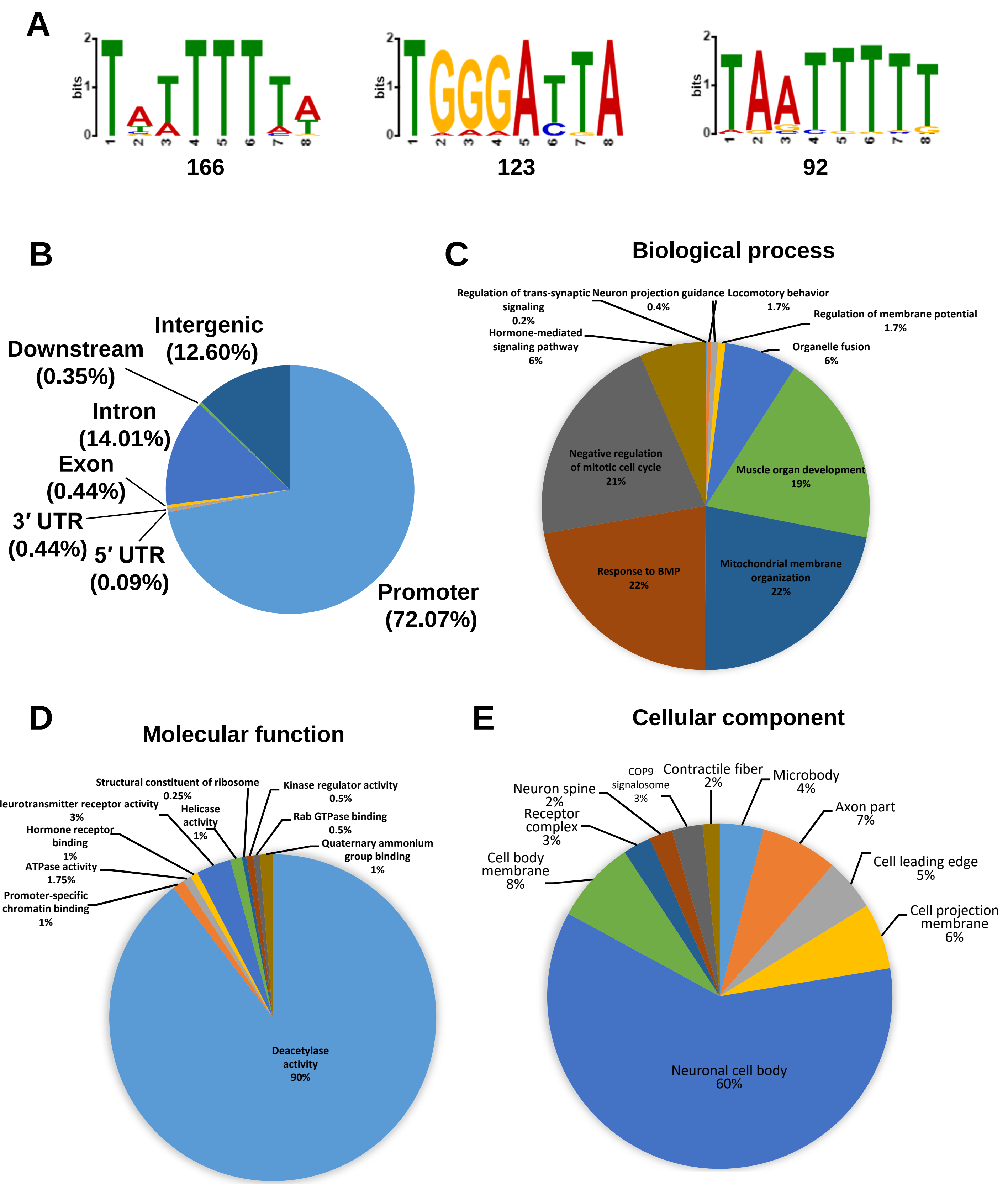

Figure S10. Identification of DDX43 KH domain bound DNA by ChIP–seq method. (A) Top three motif sequences obtained by the ChIP-Seq method. (B) Distribution of DDX43 KH domain peaks across the human genome. (C-E) Distribution of gene ontology terms among the annotated sequences for biological process (C), molecular function (D), and cellular component (E).

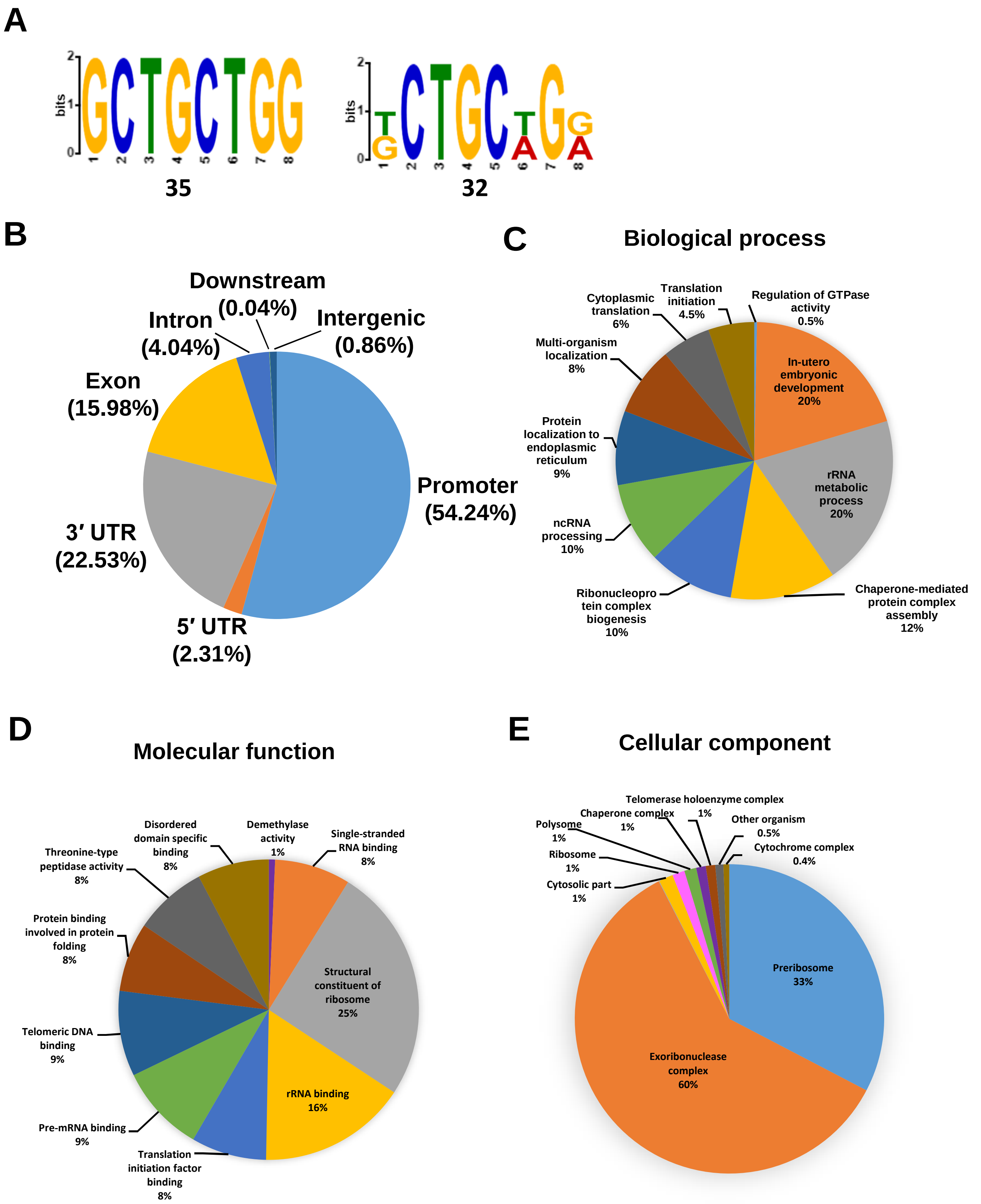

Figure S11. Identification of DDX43 KH domain bound RNA by CLIP–seq method. (A) Top two motif sequences obtained by the CLIP-Seq method. (B) Distribution of DDX43 KH domain peaks across the human genome. (C-E) Distribution of gene ontology terms among the annotated sequences for biological process (C), molecular function (D), and cellular component (E).

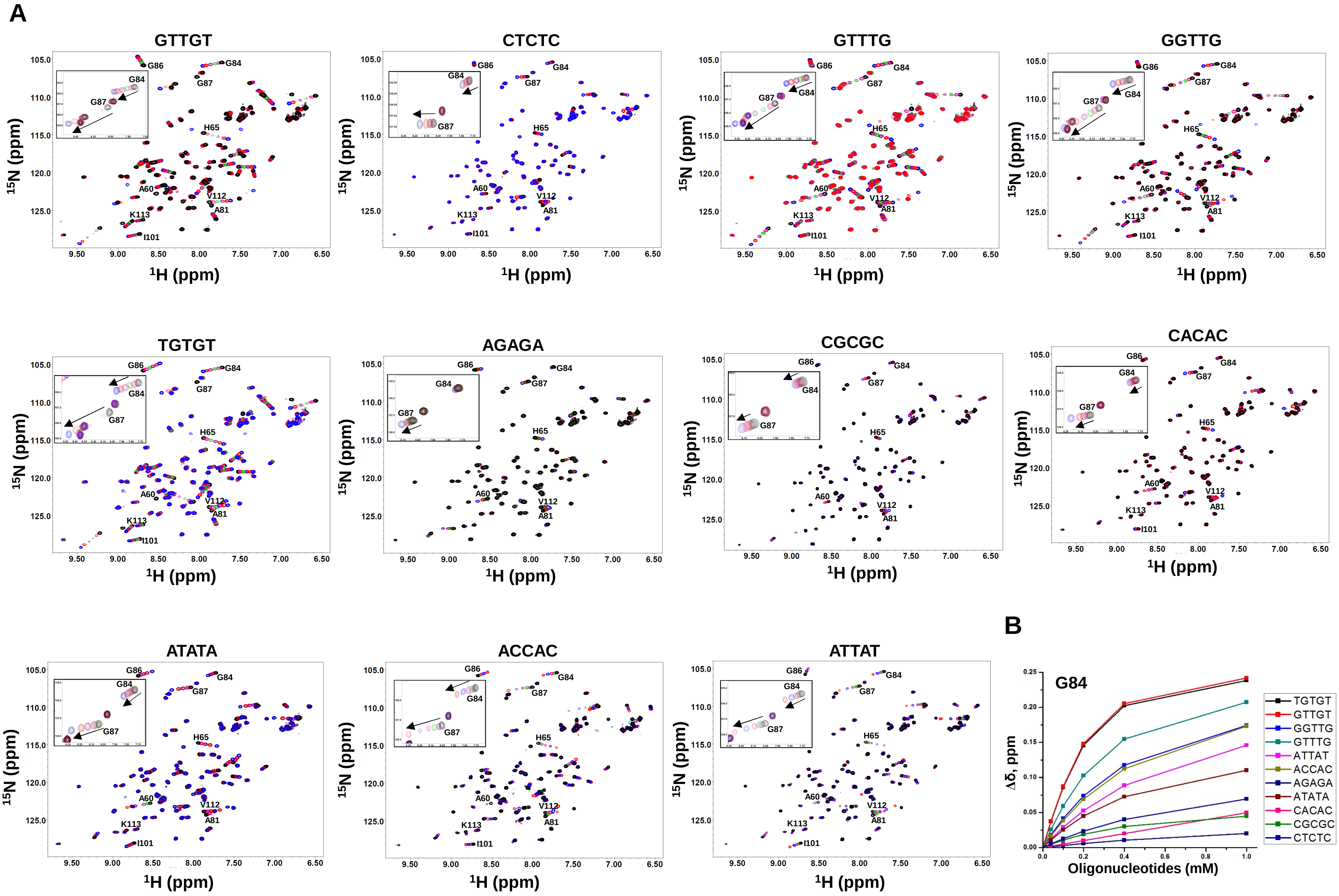

Figure S12. ^{1}H-^{15}N HSQC spectrum of the DDX43 KH-89 protein with indicated oligonucleotide (ranged 40-1000 µM). **(A)** ^{1}H-^{15}N HSQC spectrum of the DDX43-KH-89 protein (200 µM) with the indicated oligonucleotide (ranged 40-1000 µM). Insets show the enlarged view of the chemical shifts for residues G84 and G87. **(B)** Combined chemical shift change (Δδ) plotted as a function of different oligonucleotides concentration (0.0-1000 µM) at 200 µM DDX43 KH-89 protein for residue G84.

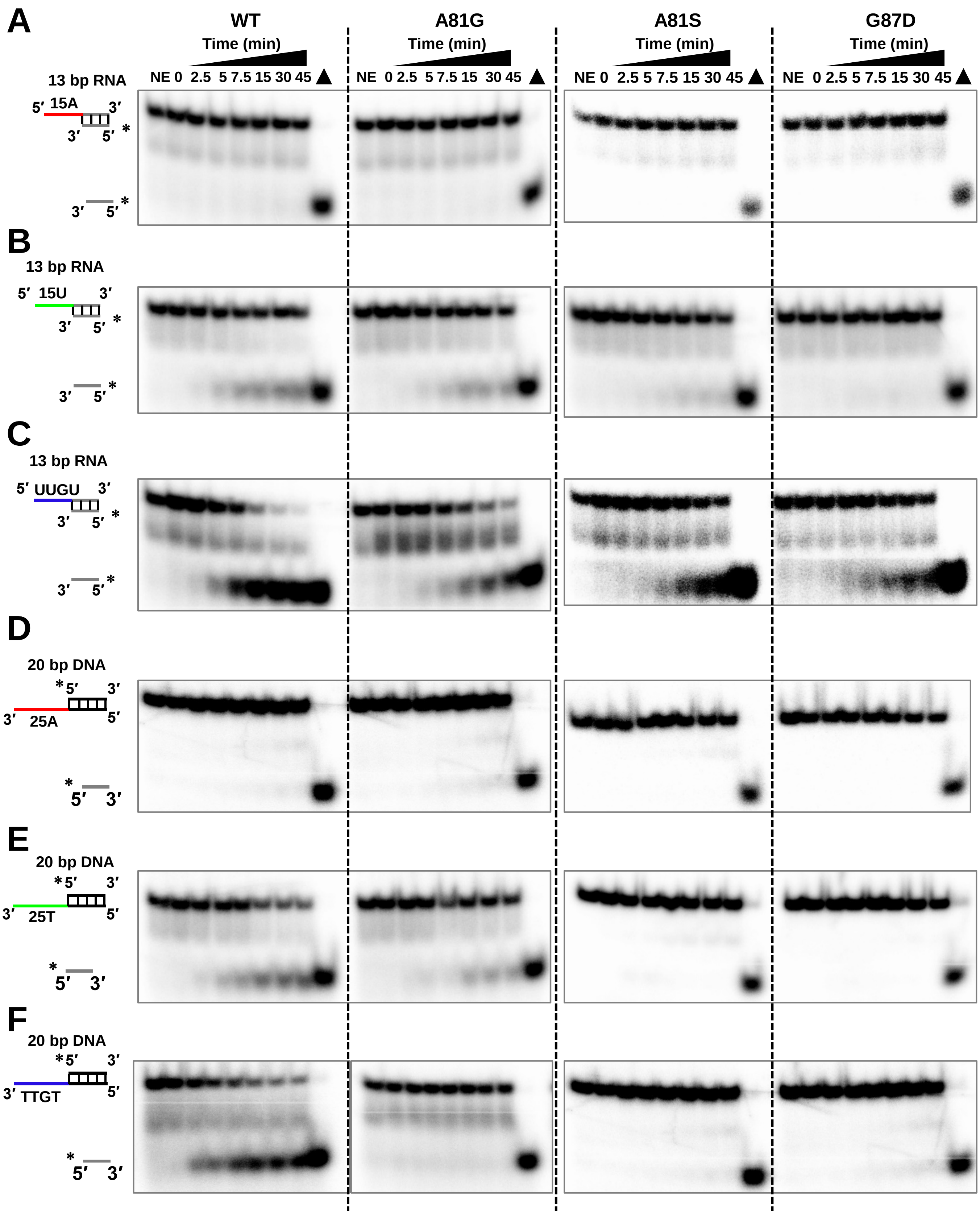

Figure S13. Representative images of helicase kinetic time course assays. DDX43 protein (WT, A81G, A81S or G87D, 2 μM) was incubated with 0.5 nM of 13-bp duplex RNA with 5′ tail of polyA (A), polyU (B), UUGU repeats (C), or 20-bp duplex DNA with 5′ tail of polyA (D), polyT (E), TTGT repeats (F), for 0, 2.5, 5, 7.5, 15, 30, and 45 min. NE, no enzyme. The triangle indicates heat denatured RNA or DNA substrate control.

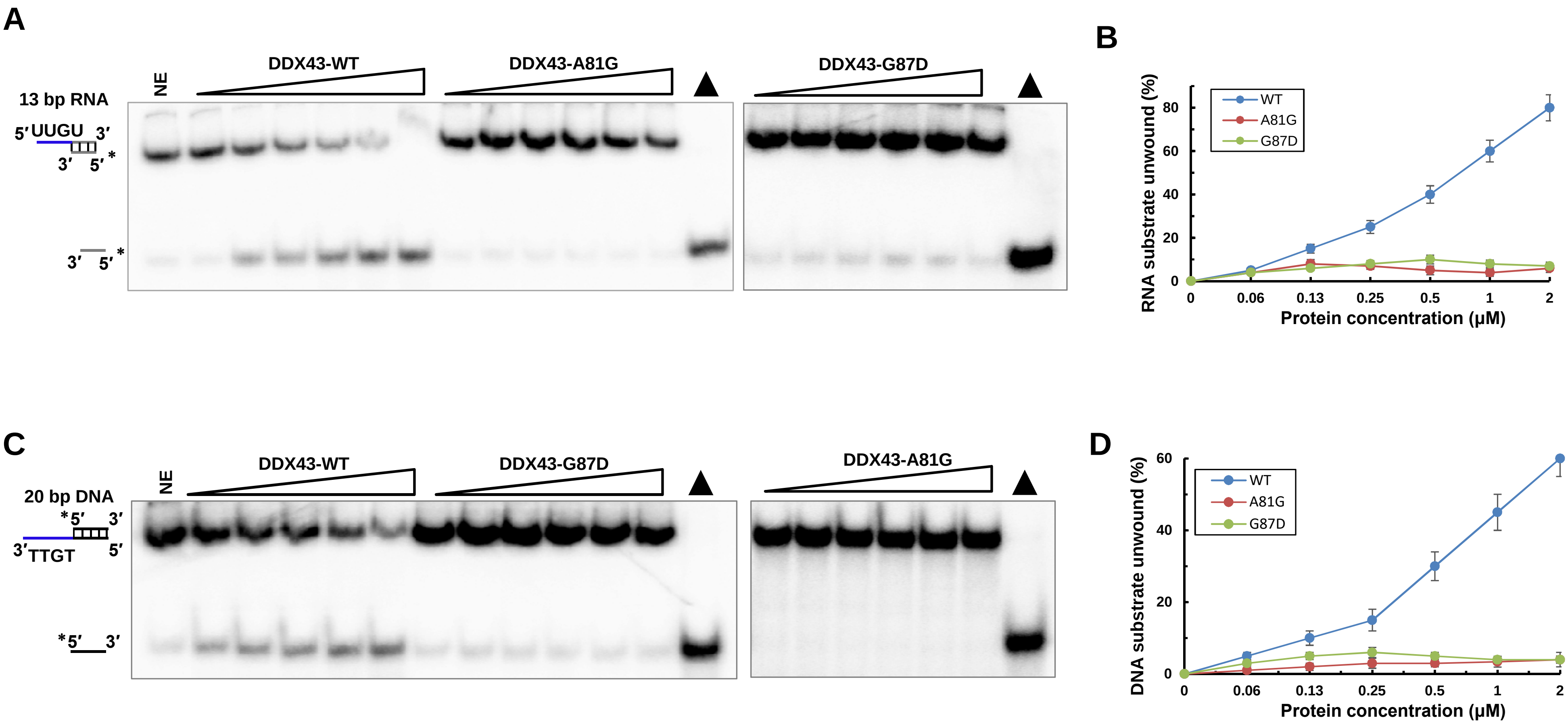

Figure S14. Mutated alanine (A81) or glycine (G87) affects DDX43 in unwinding processivity. (A and C) Representative images of helicase reactions performed by incubating 0.5 nM of 13-bp duplex RNA with 5′ tail of UUGU repeats (A) or 20-bp duplex DNA with 3′ tail of TTGT repeats (C) with increasing protein concentrations (0–2 μM) at 37 °C for 15 min. (**B and D**) Quantitative analysis of A and C respectively. NE, no enzyme; filled triangle, heated sample. Data are presented as mean ± S.D, n = 3.

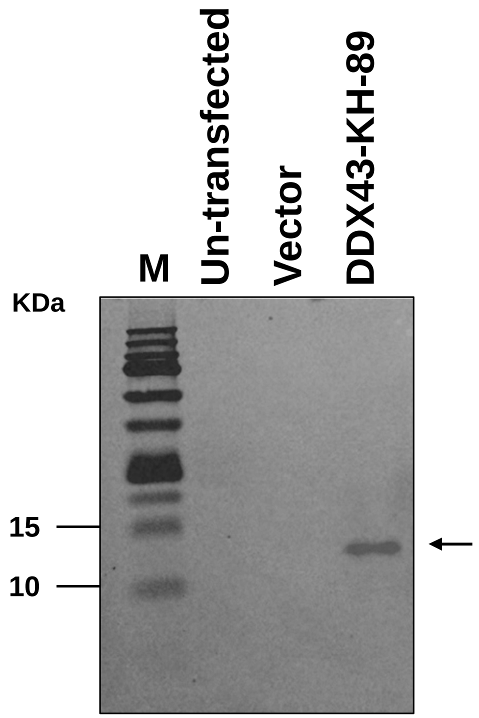

Figure S15. Western blot analysis of DDX43-KH-89 expression in HEK293T cells using an anti-FLAG antibody. M, marker.
